# Cryogenic mouse tissue homogenization as an alternative to fresh-frozen biopsy use for genomics, transcriptomics, proteomics and metabolomics

**DOI:** 10.1038/s41598-025-06438-3

**Published:** 2025-06-23

**Authors:** Laimdota Zizmare, Ute Hofmann, Mohamed Ali Jarboui, Franziska Klose, Sabine Fraschka, Jakob Matthes, Marcel Krüger, Elke Schaeffeler, Matthias Schwab, Marius Ueffing, Bernd J. Pichler, Karsten Boldt, Nicolas Casadei, Christoph Trautwein

**Affiliations:** 1 Werner Siemens Imaging Center, Department of Preclinical Imaging and Radiopharmacy, University of Tübingen, 72076 Tübingen, Germany; 2 Cluster of Excellence iFIT (EXC2180) “Image-Guided and Functionally Instructed Tumor Therapies”, University of Tübingen, 72076 Tübingen, Germany; 3 Dr. Margarete Fischer-Bosch Institute of Clinical Pharmacology, University of Tübingen, 70376 Stuttgart, Germany; 4 Core Facility for Medical Proteomics, Institute for Ophthalmic Research, University of Tübingen, 72076 Tübingen, Germany; 5 NGS Competence Center Tübingen (NCCT), Institute of Medical Genetics and Applied Genomics, University of Tübingen, 72076 Tübingen, Germany; 6 Institute of Medical Genetics and Applied Genomics, University of Tübingen, 72076 Tübingen, Germany; 7 Department of Clinical Pharmacology, and Department of Biochemistry and Pharmacy, University of Tübingen, 72076 Tübingen, Germany; 8 Core Facility Metabolomics, Faculty of Medicine, University of Tübingen, 72076 Tübingen, Germany

**Keywords:** Biochemistry, Biological techniques, Biotechnology, Molecular biology, Systems biology

## Abstract

The classical approach of using adjacent pieces of fresh-frozen tissue for various omics analysis from the same sample possesses a risk of biological mismatch between arising from intrinsic tissue heterogeneity. We propose an alternative approach of tissue cryogenic pulverization and lyophilization before distribution for omics studies for a more reliable analysis. Here, we compare individual omics layer readouts from fresh-frozen adjacent tissue pieces and homogenized powder in mouse brain, kidney, and liver. Genomics, transcriptomics, proteomics, and metabolomics analyses showed comparable RNA integrity, DNA methylation, and coverage of transcripts, proteins, and metabolites across both methods. Moreover, the homogenized-lyophilized powder usage led to reduced heterogeneity between biological replicates. We conclude that the cryogenically pulverized-lyophilized tissue approach not only maintains a critical molecular feature coverage and quality but also provides a homogenous basis for various omics analysis enhancing reproducibility, sample transport, storage and enabling multi omics base on one and the same tissue aliquot.

**Supplementary Information:**

The online version contains supplementary material available at 10.1038/s41598-025-06438-3.

## Introduction

Omics analysis has revolutionized biological research by enabling comprehensive, high-throughput investigations of molecular layers^1,2^. Most commonly employed omics—genomics, transcriptomics, proteomics, and metabolomics^3,4^—provide insight into the complex interactions and regulatory mechanisms within cells and tissues. Omics analysis have generously aided the identification of biomarkers for early disease detection, elucidated pathways involved in disease progression and highlighted potential therapeutic targets.

Moreover, single-cell omics technologies on the spatial scale have fundamentally transformed our understanding of biological complexity, enabling high-resolution mapping of cellular heterogeneity, the discovery of novel cell states, and the elucidation of lineage dynamics in health and disease^5^ in the recent years. While a rapidly developing field, single-cell techniques are not without challenges. Some limitations include restricted throughput, cost efficiency and detection of low abundant molecules, especially by single-cell proteomics and metabolomics. A comprehensive multi-omics integration across all major molecular layers within the same individual cell still remains technically challenging^6^, and therefore bulk tissue processing approaches are often still the method of choice. Established multi-layer omics studies, including single-cell approaches, often divide tissue into pieces, sections or individual adjacent cuts^7^, as each individual omics technique can have a different preanalytical prerequisite, e.g. specific tissue lysis, RNA buffer, protein digestion and stabilization, or metabolite extraction protocol. Under the assumption of generic tissue homogeneity these neighboring fresh-frozen (FF) tissue pieces then are divided and distributed for individual omics layer analysis.

First, the tissue collection quality directly affects the data reliability and therefore it is important to ensure robust collection procedures that allow deciphering the true dynamic tissue environment^8^. More importantly, when working with small animal models or precious clinical biopsies, only a limited amount of sample can be obtained, even more restricting the tissue yield available for distinct multi-layer omics distribution^9^.

Secondly, not all tissue types are equally homogenous. While some organs mayorly consist of a few cell types, others, including brain, have spatially varying compartmental composition. Moreover, the tissue heterogeneity, for example, in the case of tumor composition and microenvironment is a major reason for cancer resistance to therapy leading to adverse effects^10^. Several key genetic drivers of tumor metabolism, including genome instability and mutation, non-mutational epigenetic reprogramming, and cellular senescence^11^ have been identified, yet understanding the network and downstream cellular implications still pose a challenge to natural science and medical research fields.

This intrinsic tissue heterogeneity plays a significant role in the obtained molecular readout. Some parts of e.g. tumor or other abnormal tissue could have a more or less hypoxic, necrotic, or senescent environment, leading to a situation where different omics layers describe different nuances of the tissue environment. Therefore, only when samples originate from the same location and microenvironment, one can sufficiently link gene mutations and their RNA and protein expression with altered metabolic phenotype information levels.

When preparing multiple omics experiments, each individual omics method possesses an important and unique layer of information. Commonly used genomic techniques include panel and whole genome sequencing, and DNA methylation. As an epigenetic mechanism influencing downstream gene expression regulation^12^, DNA methylation status can reveal naturally occurring alterations, including ongoing processes involved in carcinogenesis or aging^13^.

Meanwhile, transcriptomics focuses on RNA-related aspects of gene transcription and translation^14^. There are different transcriptome study approaches focusing either on a few gene transcript elucidation (e.g. reverse transcription polymerase chain reaction (RT-PCR), 5ʹ and 3ʹ rapid amplification of cDNA-ends (RACE) quantitative (real-time) reverse transcriptase-PCR (qRT-PCR), or cDNA synthesis and Sanger sequencing) or the whole transcriptome, such as microarrays and cDNA synthesis and RNA sequencing/next generation sequencing (NGS) technology^14^.

Downstream to genomics and transcriptomics in the omics cascade, proteomics analysis is focused on not only cellular protein abundance but also post-translational modifications^15^. Proteomics is mainly performed by liquid chromatography (LC) mass spectrometry-based shot-gun approaches allowing for simultaneous large amounts of protein mass information^16,17^.

Finally, metabolomics, as the closest readout to phenotype, provides a detailed view of low molecular weight metabolites and lipids, their interactions, and perturbations within health and disease. Similar to proteomics, metabolomics largely is performed by LC- or gas chromatography MS-based analytical approaches^18^, and additionally also by absolutely quantitative nuclear magnetic resonance (NMR) spectroscopy^19–21^.

Of note, individual feature readouts obtained via omics analyses, such as DNA, RNA, proteins, and metabolites, have distinct turnover and degradation rates. Such mismatches in activity can lead to a complicated omics data correlation meaning that the abundance of a protein does not necessarily directly match its downstream metabolic turnover. For example, depending on the present enzymatic activity, the turnover rate of the amino acid glutamine can differ up to a factor of 10^22,23^. Hence, it is also crucial to consider the molecular turnover effects and normalize obtained results with caution.

Attempting more homogenized investigations, intact tissue punches have been reported for e.g. high-resolution magic angle spinning (HR-MAS) NMR-based metabolomics with further tissue pulverization for RNA microarray analysis^24^. Here, the tissue intrinsic heterogeneity issue is considered, yet particular sample handling is needed and can lead to ongoing metabolic processes. Furthermore, this approach is also then limited to only two different omics layers—the non-destructive HR-MAS and one additional destructive omics approach.

An alternative approach has been proposed in recent years aiming for homogenized biological sample preservation and analysis. Tissue cryogenic pulverization and consequent lyophilization provides an opportunity for obtaining a uniform representative specimen^25^. The water removal makes the sample not only more resistant to temperature changes but also easier to handle, store and aliquot^26^. In here, pulverized-lyophilized tissue approaches have been reported beneficial and reliable for RNA and nucleic acid long-term preservation, as well as proposed to be suitable for several omics layer analyses^25,27^.

In our study, we propose to employ the cryogenic pulverization-lyophilization approach to reduce tissue heterogeneity, that is otherwise introduced when using adjacent samples for multi-omics analysis, by an alternative preanalytical method. We aim to demonstrate how this preparation can aid tissue sample preparation for simultaneous subsequent genomic, transcriptomic, proteomic, and previously less reported metabolomic investigations. Here, we cryogenically pulverized and lyophilized (PU) freshly collected healthy mouse kidney, liver, and brain tissue, and compared their genome, transcriptome, proteome and metabolome with corresponding FF samples that were cut into individual tissue pieces.

We hypothesized that the pulverization-lyophilization approach prior the tissue distribution to various analyses will aid the dataset homogenization and provide more robust readout between the investigated individual omics layers and reduce the otherwise persisting tissue region specific heterogeneity. Furthermore, as the freeze-drying is performed and tissue is dehydrated, the tissue becomes more stable and durable for even short-term room temperature or low cooling condition storage and transport.

We employed quality control (QC) parameters to assess the turnover and degradation of RNA, proteins, and metabolites in pulverized tissue, and to compare the PU approach with the classical tissue-based FF omics analysis where an adjacent piece or section of tissue is used for each omics level. With this, we propose a robust novel method for various omics level investigation that allows obtaining more reliable molecular data for correlation and integration, based on cryogenic homogenization of the tissue prior to its distribution for omics technologies.

## Results

### Fresh-frozen (FF) sectioning and pulverized-lyophilized (PU) tissue pre-processing methods result in a comparable feature coverage

**Fig. 1:**
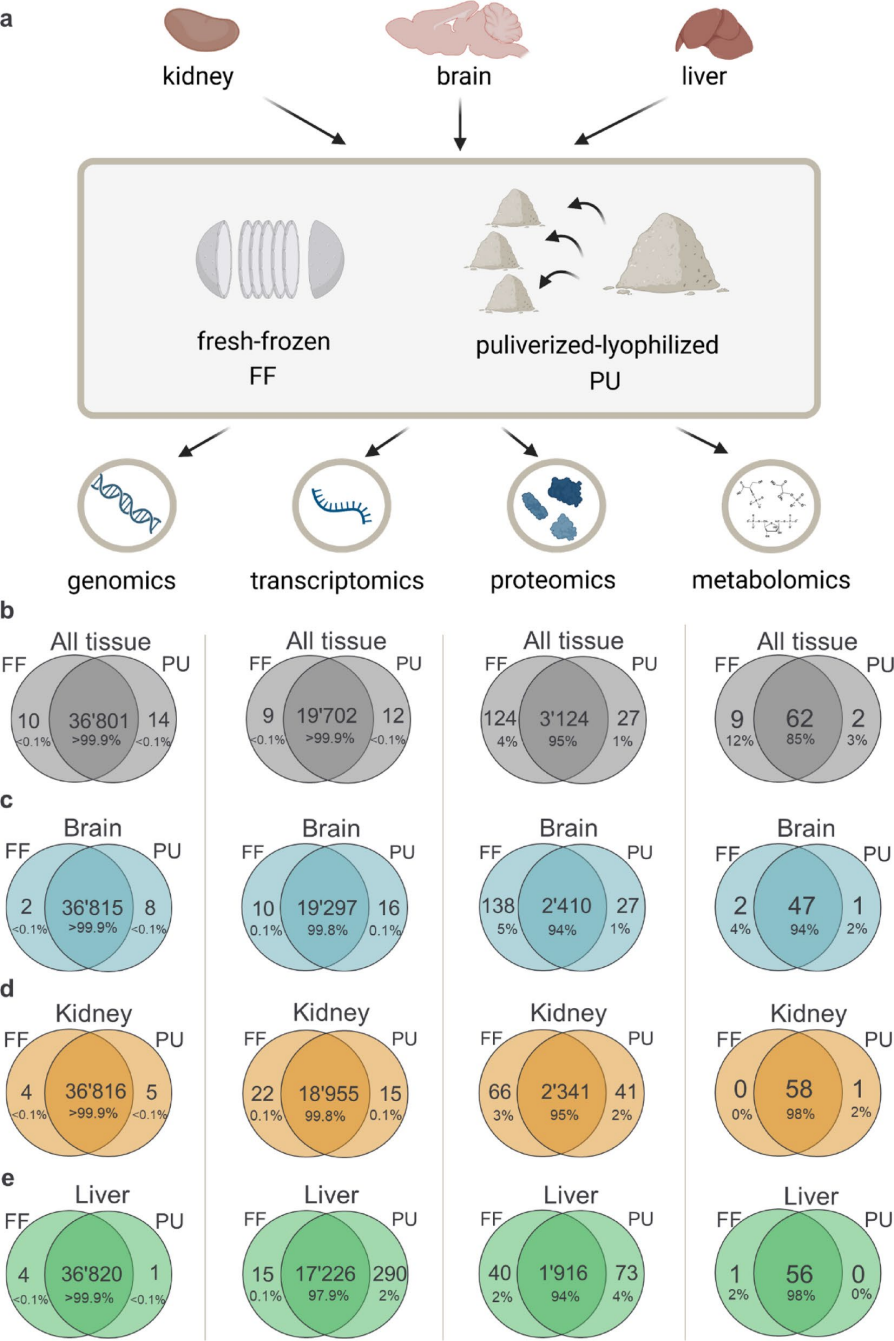
Experimental workflow setup and individual feature coverage preservation by fresh-frozen (FF) and pulverization-lyophilization (PU) approaches. (**A**) Graphical representation of the experimental workflow: healthy mouse kidney, brain and liver tissue was subjected to fresh-frozen cryogenic pulverization with and without lyophilization (n = 4) and distributed to further individual omics layer analysis. The pulverization-lyophilization approach resulted in comparable feature coverage and preservation for (**B**) all tissue types combined, and was illustrated for individual (**C**) brain, (**D**) kidney and (**E**) liver feature coverage compared with the standard FF approach.

We subjected healthy mouse kidney, brain and liver to two pre-analytical sample processing approaches: the pulverization-lyophilization (PU) and the common fresh-frozen (FF) sectioning approach prior distribution to the individual omics-layer analysis. The aim was to investigate how the pre-processing of samples compares between the currently used FF sectioning approach and our proposed new method (Fig. 1a). The initial genomics, transcriptomics, proteomics and metabolomics analysis of PU tissue resulted in a comparable detected feature coverage when comparing PU to the commonly used FF tissue processing (Fig. 1b–e).

The overall detected features in all tissue types combined resulted in 100% overlap between the PU and FF approaches in genomics and transcriptomics datasets (Fig. 1b), while proteomics and NMR spectroscopy-based metabolomics downstream datasets observed overall 85–95% features to be preserved by both methods. However, to fully conclude on the preserved feature estimation, one must consider that the genomics and transcriptomics dataset is in the dimension of tens of thousands, while proteomics feature list is just few thousand and metabolomics is below 70 features. In detail, the FF and PU similarly resulted in an overlap of 94–100% of the brain (Fig. 1c), 95–100% kidney (Fig. 1d), and 94–100% liver (Fig. 1e) features.

The homogenized PU tissue processing method is fundamentally able to equally preserve most features present in each tissue type by independent omics analyses. The lyophilization of the tissue in combination with the pulverization therefore do not negatively impact the individual omics analysis outcome.

In detail, we further report the quality assessment of individual genomics DNA methylation, transcriptomics RNA integrity, proteomics and metabolomics heterogeneity analysis illustrating the effect of the PU pre-processing method compared with the FF approach.

### Fresh-frozen healthy brain tissue has a more heterogenous pattern compared to pulverized–lyophilized samples

**Fig. 2:**
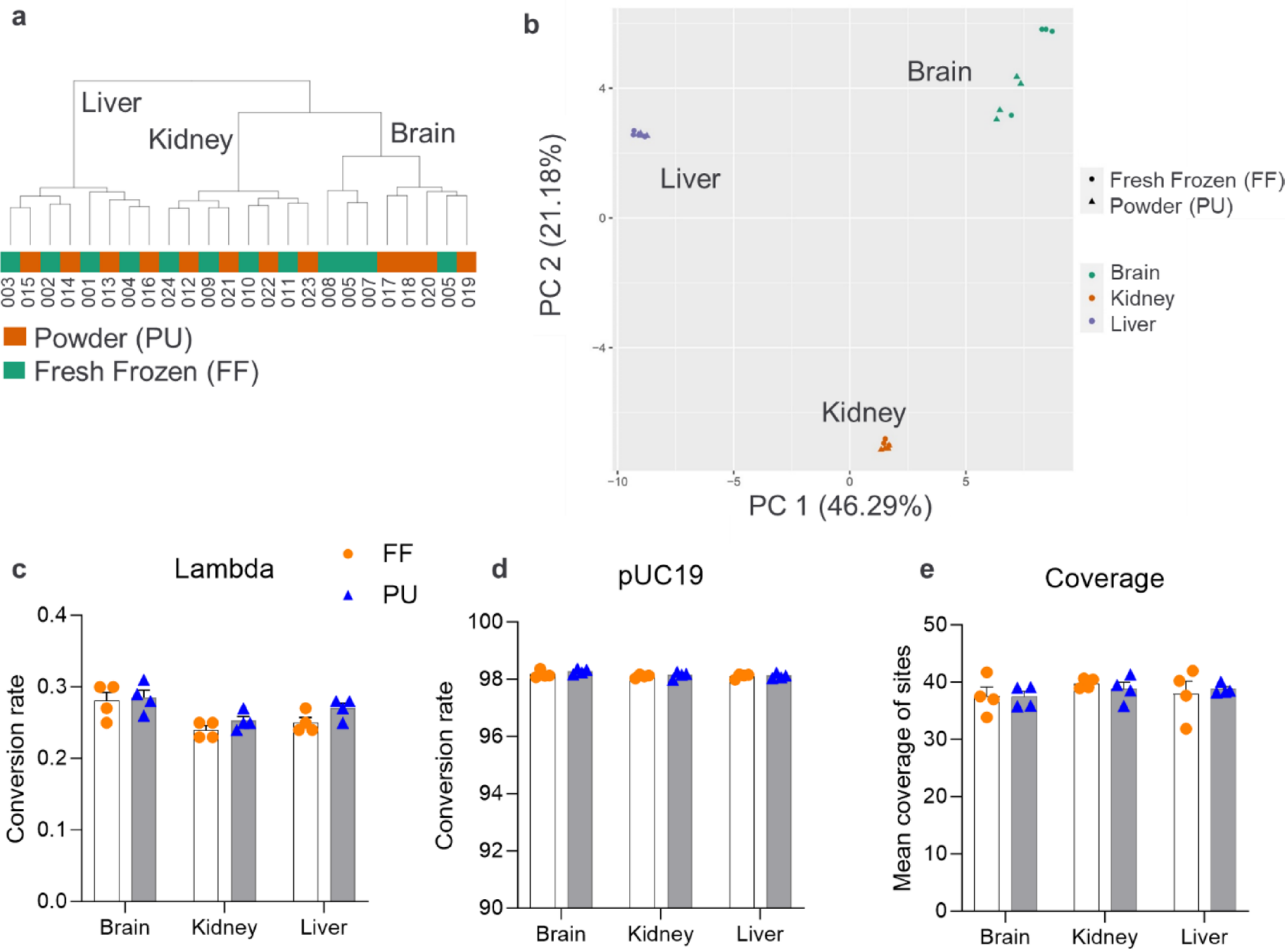
Genomics analysis by DNA methylation state of the fresh-frozen (FF) versus pulverized (PU) brain, kidney, and liver tissue. (**A**) Dendrogram visualizes the overall sample clustering within tissue types and animals, suggesting that the animal-specific genome was preserved by both FF and PU methods. Distance calculated by the Euclidean distance measure using Ward clustering algorithm and visualized as Pearson correlation coefficient. (**B**) Principal component (PC) analysis (PCA) illustrates the primary sample clustering based on their tissue type: green—brain, orange—kidney, blue—liver; dot—fresh-frozen, triangle—powder. (**C**) Unmethylated lambda control and (**D**) CpG-methylated pUC19 were spiked in to the each of the samples, and indicates specific and efficient methylation conversion with no significant differences between the fresh-frozen and pulverized-lyophilized tissue, as well as unified (**E**) genome-wide coverage of detected methylation sites. The threshold was set to one group having > 3.5 and the other < 2 mean number of samples across all considered methylation sites that have a coverage larger than 5 reads for the methylation site. Error bars represent the mean and standard error of the mean (SEM).

Genome-wide DNA methylation approach was used to investigate whether the pre-processing of samples, namely pulverizing (PU) versus fresh-freezing (FF), alone has an impact on DNA methylation profiles of murine liver, brain, and kidney samples. We applied whole-genome enzymatic methyl-sequencing (EM-seq) on biological quadruplicates (Fig. 2).

For the downstream quality control measurements, methylation status of individual tiles was analyzed across all 24 samples. We excluded all SNP-enriched sites and low-quality sex chromosome positions. In total, 479,176 tiling regions of 5000 length were kept. The hierarchical clustering analysis based on Pearson correlation distance and principal component analysis (PCA) revealed that the type of tissue, namely whether it is brain, kidney or liver, has the highest impact on the clustering (Fig. 2a, b). The highest rate of heterogeneity was observed in the fresh-frozen brain DNA methylated individual replicates, while the pulverized-lyophilized brain represented a more homogenous view between the biological replicates. This is likely to be the intrinsic heterogeneity of brain tissue which is reflected by slightly different brain area selection as biological replicates for omics analysis illustrating the need for homogenization approach for true biological alteration identification.

Unmethylated lambda control of the EM-seq^28^ presented a maximum of 0.31% conversion rate and CpG-methylated pUC19 DNA a minimum of 98% conversion rate validating the specific and efficient conversion (Fig. 2c, d). We did not observe the effect of the pulverization, of the organ, or of the individual animal on the methyl-conversion of the control spike-ins. For the murine genomes, the average genome-wide coverage ranged between 31.9 and 41.9-fold (Fig. 2e). In total, between 19.5 and 20.5 million sites were covered in all samples of the dataset (Figure S1a).

Taking together, pulverizing samples prior to library preparation does not majorly alter the DNA methylation patterns on a genome-wide scale compared to snap-frozen and subsequently homogenized samples. Meanwhile, pulverizing samples prior to performing EM-seq may help to reduce the variance between samples prepared from different batches due to a more homogeneous composition.

### Whole genome sequencing showed no general impact of different tissue preparation methods

For each tissue processing method, we performed one whole genome sequencing (WGS) analysis (Figure S1b–e). When comparing the error rates of the WGS, we obtained comparable Q scores both for the FF and the PU sample indicating generally similar coverage (Figure S1b). To estimate the quality of the preparation, DNA isolation yield was quantified. General DNA isolation yield in kidney was the most comparable between FF and PU methods, while FF liver and brain had slightly higher yields compared to the PU (Figure S1c). Meanwhile, when assessing the DNA/RNA isolation (260/280), the absorption ratio was similar between all FF and PU tissues (Figure S1d). Similarly, the DNA isolation 260/230 absorption ratio that shows the technical quality of extraction and possible contaminations showed comparable ratio for liver and kidney, while slightly higher absorption ratio was observed for the PU brain compared to FF (Figure S1e).

Together, genome-wide sequencing confirmed the comparability between the FF and PU method outcome with no major impact of the preparation method on the tissue genome and DNA methylation status.

### Pulverized-lyophilized brain tissue transcriptomics reveals reduced biological replicate heterogeneity compared with FF tissue preparation

**Fig. 3:**
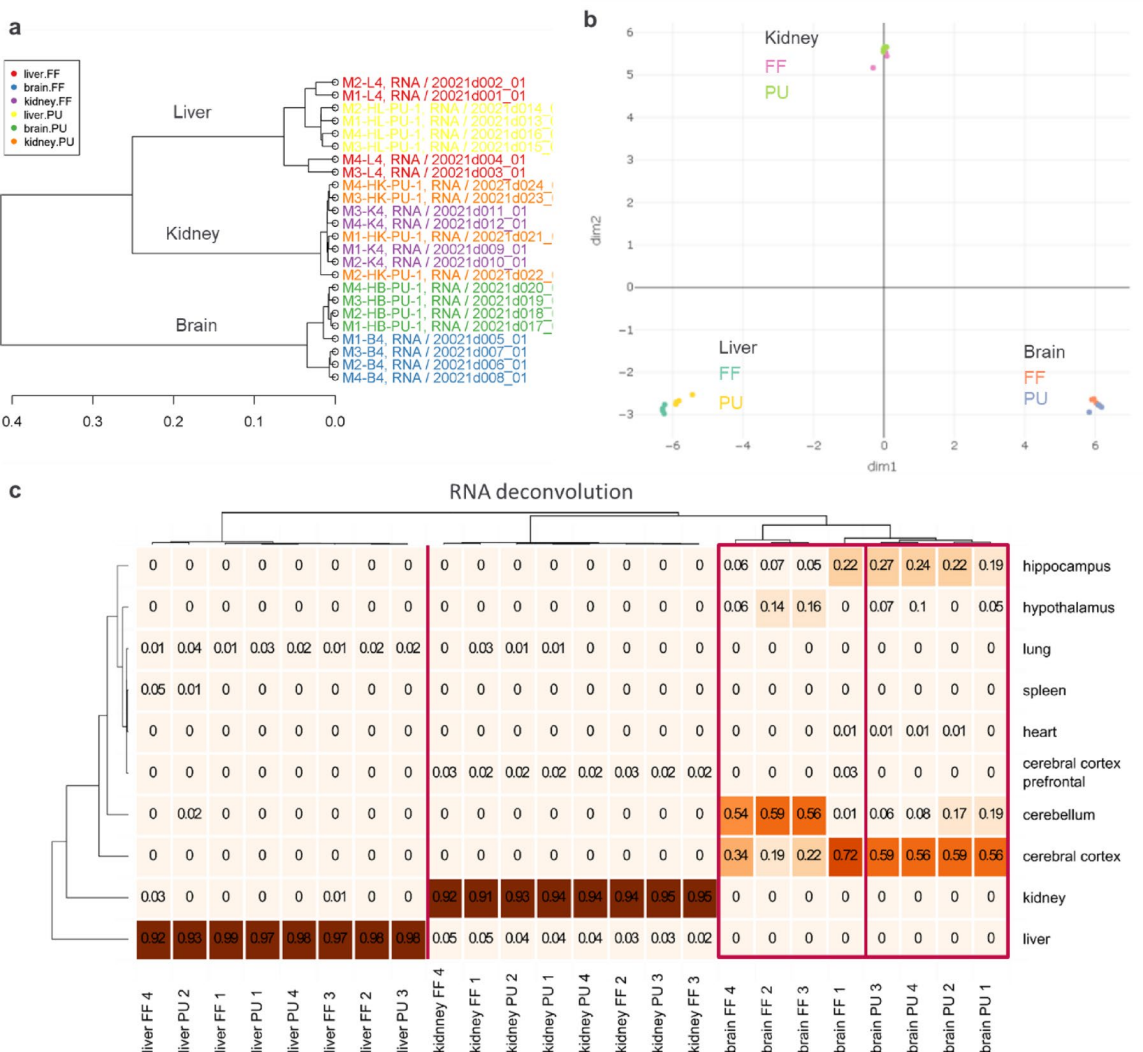
Transcriptomics reveals tissue specific heterogeneity reduction between biological replicates by pulverization pre-processing approach. (**A**) Dendrogram illustrating the Pearson r distance measure correlation for liver (FF red, PU yellow), kidney (FF purple, PU orange), and brain (FF blue, PU green). The tissue preparation method has an impact on correlation, as gene transcript preservation is connected with the homogeneity of the tissue. Distance calculated by the Euclidean distance measure using Ward clustering algorithm. (**B**) Principal component analysis (PCA) of the transcriptomics sample distribution in the first two principal components. (**C**) RNA deconvolution illustrates the impact of FF and PU approaches on organ specific heterogeneity.

When analyzing sample similarity and correlation in the transcriptomics dataset, there was no striking influence of the tissue preparation method on the kidney, but pulverized brain and liver tissue exhibited reduced heterogeneity between the tissue samples of individual animals compared with the more heterogenous fresh-frozen cohort (Fig. 3).

In the kidney, PU (orange) versus FF tissue slicing (purple) had the least effect, illustrated by mouse 1 (M1) and mouse 2 (M2) sample closest correlation despite the sample processing method (Fig. 3a). Meanwhile, FF liver (red) had rather different gene transcript readouts compared to PU liver (yellow). General tissue separation based on principal component analysis was mainly tissue-type based, with larger replicate separation in FF compared to PU brain and liver (Fig. 3b, Figure S2j–l).

Interestingly, when observing the RNA deconvolution (Fig. 3c), sample homogenization approach (PU) showed the most benefit in brain tissue illustrating reduced heterogeneity between the individual replicate brains while FF brains exhibited individual variability depending on the randomly selected brain sliced region, that could potentially lead to false interpretation of overall tissue transcriptome and unfavorable outcome for further data integration approaches.

Differential gene expression was further investigated in the context of pathway analysis based on the IPA the logFC of genes. With a threshold of |FC|> 1 and FDR < 0.05 we observed dysregulated 5414 genes in the liver and 527 dysregulated genes in the brain, while kidney did not have any strongly regulated genes within the set threshold. Moreover, the dysregulation in almost all of the pathways was not specific to the tissue preparation method as FF or PU, and the pathway enrichment was rather random (Table S3).

When reducing the threshold to |FC|> 1 and p < 0.01, kidney presented 64 dysregulated genes, 947 genes in brain, and 4957 dysregulated genes in the liver. We concluded that the overall FF versus PU tissue analysis did not identify a direct effect of the pre-processing method related to a specific pathway alteration. For example, the cAMP response element-binding protein (CREB) CREB signaling pathway in neurons was one of the most dysregulated in all three tissue types, however, while it was upregulated in FF liver and brain, in FF kidney the pathway was downregulated compared to the PU tissue (Table S4).

Overall, the transcriptomics dataset provided a comprehensive overview showing only very marginal alterations related to the FF or PU tissue preparation, and indicated a high degree of similar transcript preservation by the PU compared to the FF. Transcriptomics also highlighted the benefit of tissue homogenization leading to reduced biological replicate heterogeneity in brain.

### The pulverization–lyophilization method had no negative impact on the yield, quality, and integrity of RNA

We also assessed the isolated RNA quality used for the comparative analysis to investigate whether the pre-processing of samples, PU versus FF alone, has an impact on genome-wide gene expression profiles of the murine liver, brain, and kidney samples.

The sample pre-processing method showed no major impact on the yield of isolated RNA, yet, RNA yields varied tissue-dependently (Figure S2a–c). Statistically, 2-way ANOVA revealed that tissue type was the main differentiating factor for RNA integrity (p = 0.0055) and RNA yield using photometry and fluorescence measurements (p = 0.008 and p = 0.0098, respectively), while the tissue processing method was not significantly different for brain, kidney or the liver tissue. Brain and kidney RNA integrity and yield were the most consistent for both PU and FF approaches, whereas liver samples presented inherent variability likely related to the initial sample collection.

The RNA quality was assessed using the RNA integrity number (RIN) when determined with the Agilent 2100 Bioanalyzer System algorithm, which assigns a number to the level of RNA degradation of a sample based on the analysis of the area before the 18S rRNA peak, the area of the 18S and 28S rRNA peak and the ratio of 28S and 18S peak. The RIN number directly correlated with the tissue type, indicating the best RNA integrity for the brain, followed by the kidney and liver with the lowest RNA integrity, but showed no clear correlation with the freezing method (Figure S2d). Regarding the RNA purity, the 280/260 ratio seemed to be rather tissue-dependent than method-dependent (Figure S2e). For the 260/230 ratios, no clear trend was visible, however, the brain powder samples gave the lowest 260/230 ratios on average (Figure S2f). In summary, the yield, purity, and integrity of the isolated RNA varied tissue-dependently but not due to the tissue preparation method, suggesting that the PU approach is able to preserve RNA equally well compared to the FF approach.

### The type of tissue preparation did not impact the performance of the library preparation

We then monitored the impact of the tissue preparation method on the performance of the library preparation (Figure S2g–i). We did not observe any measurable impact of the tissue preparation method or tissue on the fragmentation output. The yield of the library preparation was also not significantly modified by the tissue preparation method but was influenced by the tissue type, however, we observed slightly lower library concentration in the FF cohort compared to the PU (Figure S2g). The FF presented generally higher library concentration, likely due to a better nuclein quality resulting in more efficient amplification or index ligation. Of note, DNA sequencing results did not suggest that pulverization could lead to nuclein changes affecting DNA sequencing.

Interestingly, we could detect that both, tissue type and preparation method, influenced the performance of the index ligation and therefore the amount of index primer dimer found in the final library (Figure S2h). After complimentary clean-up, we did not detect an influence of the tissue preparation method on the number of clusters generated per sample (Figure S2i). In addition, we compared FF and PU impact on transcriptome via pathway enrichment analysis. The liver transcriptome resulted the most changed, which could be explained by the larger variability between the extracted RNA concentration within the biological replicates, while the brain gene ontology biological processes showed only two mildly altered pathways (Figure S2m–n). The kidney pathway enrichment analysis resulted in no hits indicating no significant pathway changes when comparing FF and PU approach impact on transcriptome.

### Proteomics confirms a similar protein coverage preservation by both pre-analytical PU and FF approaches

**Fig. 4:**
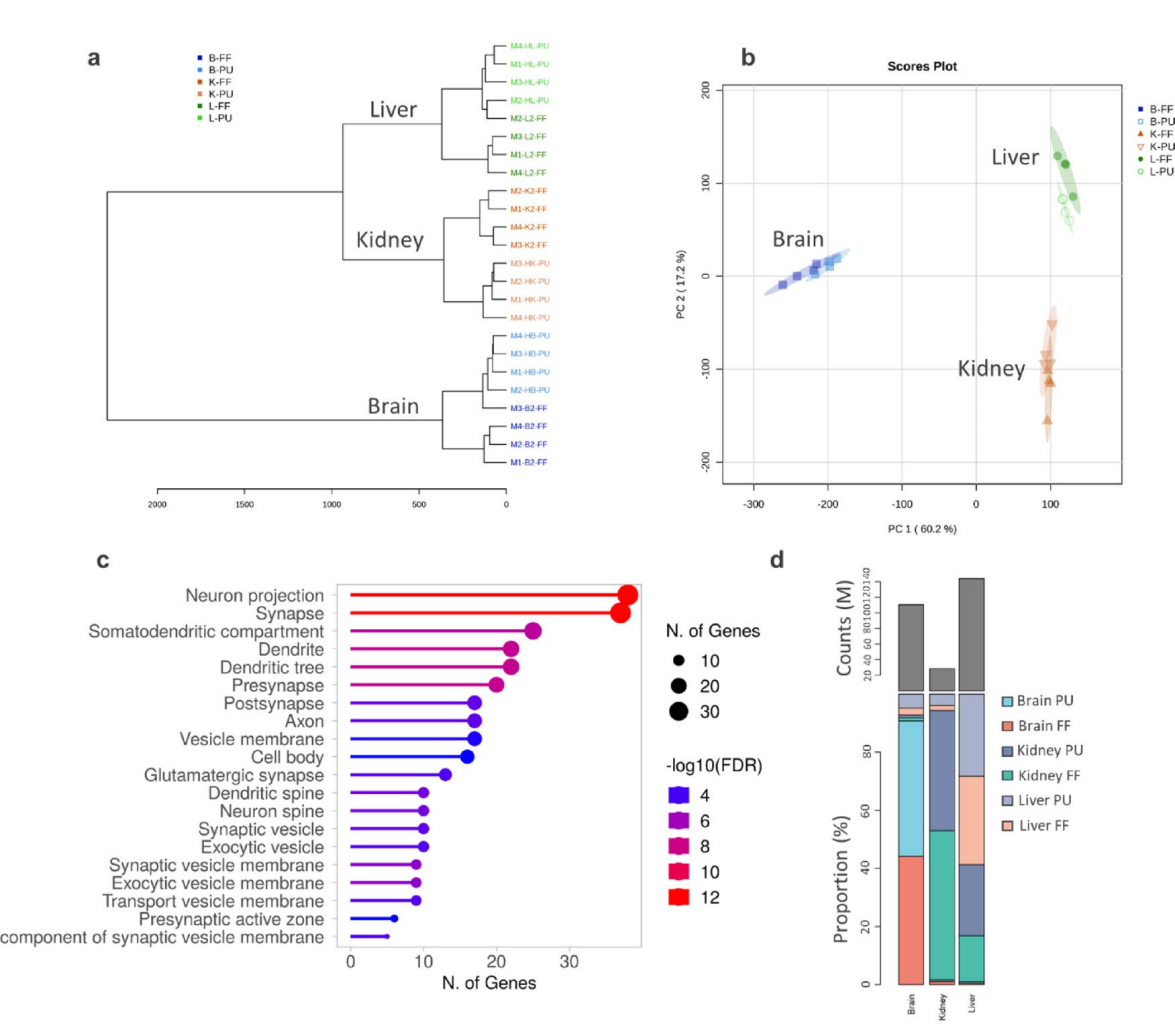
Assessment of the pulverization-lyophilization impact on the protein analysis. (**A**) Dendrogram of pulverized-lyophilized (PU) tissue compared to fresh-frozen sliced (FF) tissue intercorrelation. (**B**) Principal component analysis of all three tissues illustrating tissue-specific clusters. (**C**) The elucidation of all tissue type combined analysis of FF method impact reveals mainly affected neuronal function-related pathways based on GO biological process pathway database. (**D**) Protein coverage illustrated in relation to protein counts and the proportion of reads specific to FF and PU brain, kidney and liver.

Similarly, proteomics was investigated to determine the PU pre-analytical method impact on the proteome (Fig. 4). The PU method resulted in closer correlation and more similar proteomic outcome, visualized in the dendrogram (Fig. 4a).

Some fresh-frozen sliced tissues, such as the brain (M3) and liver (M2) correlated closely with the pulverized tissue rather than other FF from the same tissue type, illustrating the homogenization effect of the PU tissue approach compared to fresh-frozen tissue slicing. Besides the tissue-specific correlation in the PCA (Fig. 4b), reduced proteome variability was observed in the individual organ-specific PCAs when the tissue was homogenized, compared to the direct tissue slicing (Figure S3 a-c).

As all tissue types together had 95% overlap of the detected proteins (Fig. 1), the 5% of unique protein variability mainly arose from brain tissue origin specific proteins in FF tissue. 27 individual proteins in PU did not correlate to a specific functional pathway (Fig. 4c, Figure S3d, Table S5).

The overall proteomics quality was assured and estimated by analyzing the overall proteome coverage counts and proportion of proteome associated with the individual brain, kidney and liver tissue (Fig. 4d). The kidney proteome counts were lower compared to those of brain and liver. Proportionally, the kidney had the highest specific protein coverage with 97% unique proteins, similar to brain tissue having about 95% only brain specific proteins. The liver tissue contained about 60% of liver specific proteins with additional about 38% of kidney-related proteins.

Considering the quality of protein group coverage, overall fresh frozen liver and brain resulted slightly lower counts below 3000 compared to the pulverized counterpart with average of > 3000 counts (Figure S3e). Fresh frozen or pulverization-lyophilization did not directly affect the kidney protein count. In addition, comparable protein coverage with about 49–52% proportion were obtained from both FF and PU (Figure S3f). We conclude that the pre-analytical pulverization-lyophilization result in similar protein coverage compared to the fresh frozen slicing approach. Yet, the PU showed similar biological replicate heterogeneity in kidney, yet improved reduced heterogeneity in liver and brain compared to FF.

### Quantitative and targeted metabolomics shows comparable energy charge

The low-molecular weight compound investigation was performed by both LC–MS and NMR spectroscopy-based metabolomics. While the targeted LC–MS approach elucidates specific metabolic pathways of interest in detail, such as glycolysis, with a nearly complete pathway-specific metabolite coverage, the NMR spectroscopy approach provides a broader quantitative overview of a wider range metabolites in various pathways.

**Fig. 5:**
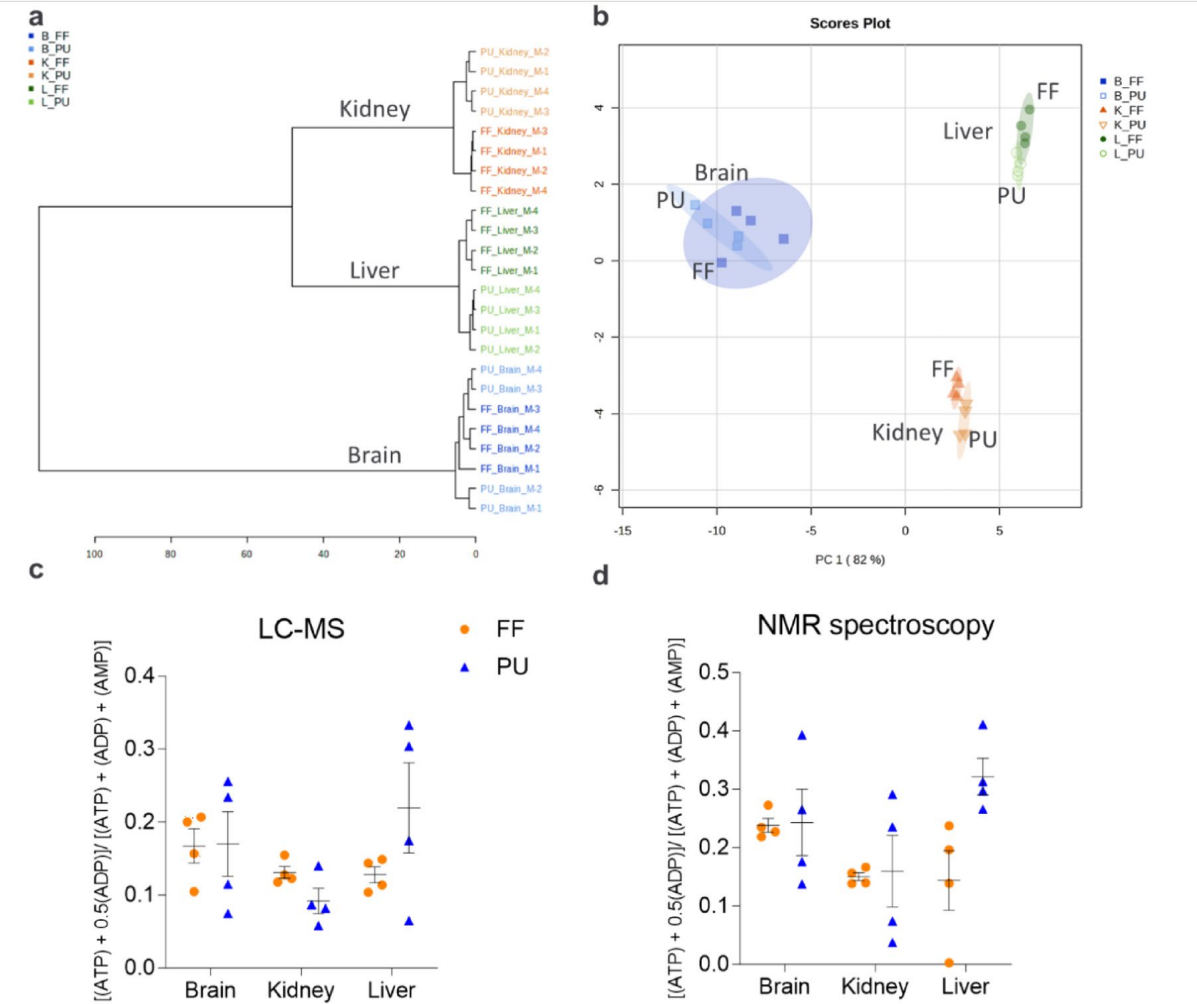
Fresh-frozen (FF) and pulverized (PU) brain, kidney, and liver metabolomics. (**A**) Dendrogram illustrating metabolite concentration homogeneity in the pulverized–lyophilized samples compared to fresh-frozen adjacent cryo-sectioning by NMR spectroscopy-based metabolomics analysis. Distance calculated by the Euclidean distance measure using Ward clustering algorithm. (**B**) Principal component analysis (PCA) of brain, kidney, and liver sample separation based on metabolite concentrations show improved heterogeneity of biological tissue replicates for PU. Calculated adenylate energy charge based on (**C**) targeted LC–MS and (**D**) NMR spectroscopy-based metabolomics shows similar average trend for both FF and PU approaches, independent of the measurement method and the tissue processing approach. No significant difference by one-way ANOVA. Error bars represent the mean and standard error of the mean (SEM).

In line with the transcriptome and proteome, metabolome revealed tissue-specific correlation patterns (Fig. 5a, b, Figure S4). Moreover, the visualization of the individual samples by organ-specific PCAs showed a reduced replicate heterogeneity in brain and liver PU samples compared to the FF counterparts (Figure S4a–c). Correlation analysis illustrated persistent correlation patterns between the PU tissues likely due to reduced heterogeneity between the PU samples, which is generally expected when tissue is prepared for analysis in the FF manner (Figure S4d).

We then investigated sugar phosphates in detail as intermediates of glycolysis and pentose phosphate pathway (PPP). These tightly connected pathways play an essential role in cellular energy metabolism. Of note, sugar phosphates can be highly sensitive to pre-analytical sample handling. The sum of sugar phosphates related to glycolysis and PPP, glucose-6-phosphate (Glu6P), ribose-5-phosphate (Ribo-5P), sedoheptulose-7-phosphate (Sed7P), ribulose-5-phosphate (Ribu-5P), 6-phosphogluconate (6-PGluc), 2/3-phosphoglycerate (2/3-PG), phosphoenolpyruvate (PEP), and fructose-1,6-bipshosphate (F-1,6-BP) as the ∑ glycolysis and PPP metabolites indicated that pulverization–lyophilization approach preserved relatively higher concentrations of the sugar phosphates in brain and liver, compared to the FF tissue. Meanwhile, kidney ∑ glycolysis metabolites in PU were slightly lower, yet comparable with the FF tissue (Figure S4e).

Both NMR spectroscopy and targeted LC–MS-based metabolomics approaches complement and validate the findings inter-methodologically. The adenylate energy charge was calculated based on adenosine mono-, di-, and triphosphate (AMP, ADP, ATP) following the formula: ([ATP] + ½ [ADP])/([ATP] + [ADP] + [AMP])^29^. The obtained energy charges were generally low starting from 0.05–0.10 to a maximum of 0.25–0.40 (Fig. 5c, d). Importantly, the energy charge of both metabolomics approaches provided a similar outcome: the mean brain and kidney energy charge was similar for the FF and PU tissues, while liver FF energy charge was lower compared to the PU method. Hence, in the case of liver, the pulverization-lyophilization approach could be beneficial for long-term adenosine mono-, di- or triphosphate species preservation. Of note, the energy charge variation from sample to sample was relatively large, excluding the possibility of observing a statistical significance (Fig. 5c, d).

## Discussion

In the light of recent developments and advances in spatial single-cell omics approaches, bulk tissue multi-layer omics analysis remains a foundational, affordable, and complementary approach in systems biology, offering comprehensive insights into tissue-wide molecular landscapes. Here, we propose an improved pre-analytical method for simultaneous subsequent omics investigations of tissue samples and show that this approach not only results in a similar data quality and feature coverage but also adds a beneficial aspect of reduced heterogeneity that is an existing limitation when using adjacent tissue pieces for individual omics analyses.

Healthy mouse brain, kidney and liver tissue were used to establish a reliable baseline to further investigate the impact of pre-analytical tissue processing on selected omics layers. Cryogenically pulverized (PU)–lyophilized tissue was compared to fresh-frozen (FF) and adjacent tissue cuts which is a commonly used alternative approach for bulk tissue multi-omics.

The main drawback of the established FF approach is that the adjacent cut pieces of preclinical tissue and small clinical biopsies often are considered homogenous. Yet, some organs, including brain, are made up of diverse cell types, or a disease like cancer is very heterogenous and not symmetrical, which leads to different tissue region investigation by various individual omics layers.

Single-cell technologies have revolutionized our understanding of cellular diversity, enabling the dissection of complex tissues and the identification of novel cell types and states^5^. These approaches are particularly valuable in contexts where intracellular heterogeneity plays a critical role in disease progression and treatment response.

However, single-cell methods often involve complex sample preparation, higher costs, and limitations in capturing low-abundance biomolecules, that can be justified depending on purpose and research question. While the single-cell field advancements have brought game-changing access to biological information at a cellular-resolution, currently covering genomics, transcriptomics, proteomics and metabolomics in one and the same single cell with multi-omics layer integration is still challenging^30^.

In contrast, bulk tissue homogenization allows for the comprehensive analysis of DNA, RNA, proteins, and metabolites from a homogenized sample, providing an averaged molecular profile that is highly reproducible and less susceptible to sampling bias. This keeps bulk processing approaches attractive for large-scale projects^31^, including population-scale studies and clinical biobanking, where consistency, cost-effectiveness, scalability, and throughput are paramount.

For example, additional separation methods, such as chromatography in LC–MS-based metabolomics and proteomics, homogenized tissue sample analysis currently results in the identification of more features with improved sensitivity when compared to spatial metabolomics or proteomics. Considering genomics and transcriptomics, single-cell DNA analysis is currently performed only on specific panels of genes and a specific design for fusion genes at the RNA level^32^.

Also, with limited tissue amounts commonly acquired from a mouse model or tumor metastasis dissection, the overall amount tissue might be too low to split it into enough individual sections for all omics investigations. Therefore, using adjacent omics information within an integrated multi-omics correlation poses a potential risk of attempting to link a diverse cohort of bio-molecular events that are not actually arising from the same exact location. It is crucial that samples considered for linking gene mutations with relevant altered metabolic phenotypes originate from the same tissue subtype and location.

Other tissue pre-processing approaches for multi-omics that report employing cryogenic pulverization while maintaining tissue water content, relay on extensive temperature control protocols^33^. Therefore, we propose that the cryogenic pulverization and lyophilization approach provides a homogenous and stable sample leading to representative and reliable multi-layer omics analysis. To assess this, a comprehensive various omics layer analysis was performed with the assessment of tissue quality comparing PU versus the FF approach.

### FF and PU both lead to comparable DNA and RNA turnover and degradation

DNA methylation is one of the key quality parameters investigated by the genomics approach^34^ as it provides an important view of ongoing biological changes over a lifetime. DNA methylation, the methylation of the nitrogenous base cytosine forming 5-methylcytosine (5′-mC), is primarily found at CpG dinucleotides. It is one of the major epigenetic modifications of mammalian genomes which, together with other epigenetic factors, plays a crucial role in cell fate commitment and differentiation processes during development. Also, DNA methylation alterations contribute to the development of various types of human cancer. Often, differing DNA methylation patterns can be observed at specific genomic loci when comparing cancer and normal tissue.

The PU tissue processing provided a comparable DNA methylation readout to the one obtained from the widely used fresh-frozen tissue multi-omics. Not only does the proposed method provide equal readout, but also the PU approach could serve as perquisite for improved batch-to-batch comparison and reproducibility. Moreover, the RNA integrity was also preserved by the PU method when compared to the FF tissue RIN, the numeric measure for RNA integrity^35^. Therefore, we conclude that the DNA and RNA turnover and degradation by the PU method is comparable to the established FF approach.

### DNA methylation, RNA transcript, protein, and metabolite detection coverage and feature preservation are not affected by lyophilization

We evaluated the coverage of features that can be obtained by different omics cascade layers. Overall DNA methylation state did not significantly vary between both methods, confirming that PU approach does not affect the DNA methylation similar to the widely established FF approach.

RNA isolation from brain, liver, and kidney tissue was tissue-type dependent. Brain sample RNA integrity was the most consistent, while liver and kidney samples displayed greater variability, with liver samples showing particularly pronounced inter-sample differences. However, our current findings do not provide sufficient evidence to link this interaction to a specific biological phenomenon. The high variability observed in the liver samples further complicates the result interpretation. These findings further highlight the necessity to consider tissue-specific factors and freezing techniques when developing RNA isolation protocols for studies involving multiple organs.

When considering transcriptomics (RNA-seq) obtained features of gene transcripts, both FF and PU methods resulted in similar feature coverage. When looking at an individual tissue type in detail, only small differences could be observed. From about 17–20 k characterized gene transcripts, 0.1% of unique features varied between the PU and FF brain and kidney. Pulverized-lyophilized liver tissue could preserve 2% unique gene transcripts compared to 0.1% of FF, likely due to better amplification and ligation of higher quality RNA obtained.

Similarly, from two thousand characterized proteins, all three tissues resulted in about 95% similar protein feature coverage. For the brain, kidney and liver individually, the PU and FF methods preserved comparable numbers between 1 and 5% unique proteins.

Meanwhile, two targeted metabolomics approaches resulted in almost identical feature coverage in all three tissue types. Depending on tissue type, only 1 or 2 individual features were unique to PU or FF method by the NMR spectroscopy-based 50–60 metabolite quantification. With this we concluded that the specific method of tissue pre-processing (PU or FF) does not have a general impact on the number of metabolomic features obtained. Of note, tissue pulverization is already employed in various NMR spectroscopy-based metabolomics studies as the pre-analytical metabolite extraction protocols require homogenized power. Similar lists and the number of quantified metabolites are reported in preclinical and clinical brain^36,37^, kidney^38,39^, and liver^40,41^ literature, compared to the ones in this study.

Overall, all employed omics in this study showed a generally good agreement between the PU and FF methods and demonstrated their ability to equally preserve majority of layer-characteristic features. In specific tissues, for example brain, that possess intrinsic heterogeneity, pulverization-lyophilization approach resulted in more homogenous readout increasing the reliability of finally obtained multi-omics data connectivity.

### Energy charge and altered metabolic pathways show a similar readout for both FF and PU derived data

Both NMR spectroscopy and MS-based metabolomics approaches provided quantitative data for the adenosine nucleotides ATP, ADP, and AMP. Based on this information, the adenylate energy charge (AEC) could be calculated^29^. When the cellular system is out of charge, the energy charge is zero, while depending on the balance, a fully charged system would result in an index close to one^42^. Typically, most cells possess an energy charge of around 0.6 and higher^29,42^. Based on the calculation in this study, the obtained energy charge was generally low, below 0.4. This could be potentially due to the fast-molecular turnover and dynamic interplay of adenosine nucleotides when tissue processing, prolonged storage, and evaluation are performed ex vivo. Furthermore, the relatively long-time spans between sacrificing the mice and quenching the organs in liquid nitrogen must be considered as reducing factor of AEC values.

The data obtained by NMR spectroscopy and MS provide visually similar energy charge trend validating the results obtained as method unspecific. Also, the PU and FF methods show comparable energy charge preservation in the brain and kidney, while in the liver PU method has a trend of higher energy charge compared to the FF. Energy charge results suggest that not only does the pulverization-lyophilization tissue pre-processing yield a comparable energy charge, but also that in different tissues, e.g. the liver, the PU method could be potentially more effective for tissue energy charge preservation.

Furthermore, when assessing specific common metabolic pathways, such as glycolysis and PPP, the sum of sugar phosphates involved in these pathways had an increased trend in the PU tissue. Findings suggest that the pulverization-lyophilization approach can not only provide comparable metabolic preservation but also be somewhat beneficial as seen on an individual pathway level.

### Use of lyophilized pulverization aliquots improves biological replicate heterogeneity in the omics landscape

We further investigated the PU and FF tissue processing method impact on the overall individual replicate heterogeneity. Confirming our hypothesis, the PU method resulted in generally reduced heterogeneity between the individual replicates within each group of individual tissue type. The genomic, transcriptomic, proteomic, and metabolomic principal component analysis, dendrograms, and feature correlation maps showed generally closer clustering of the PU tissue samples than the FF tissue samples providing the main indicator of reduced replicate heterogeneity by the PU approach. The reduced replicate heterogeneity is a positive outcome also because of the general need for further reliable multi-omics layer integration. The less individual replicates vary among themselves, the more trustable multi-omics-layer integration becomes.

Some of the limitations of this study include the fact that the tissue homogenization approaches as well as adjacent tissue cuts subjected to bulk multi-layer omics analyses averages the molecular content across all cells and types within the specimen. Hereby, cell-type-specific and cell-to-cell multi-omics-layer variation information is inevitably lost, which removes the spatial context. While it is true that single-cell methods can explicitly characterize cell-to-cell variability, bulk analysis provides an essential and relevant information about population-level regulation, including consensus regulatory networks, pathway-level changes, and robust biomarkers. Depending on the biological context where the goal is not to capture variance but to identify consistent, tissue-level shifts, bulk analysis persists as relevant and viable approach also for low-abundant features.

Moreover, a further comparison of the multi-omics data integration is limited and spanning beyond the scope of the initial work of individual omics layer evaluation. However, the observed individual omics layer information suggests that reduced heterogeneity may be further beneficial for multi-omics data integration and reliable interpretation, considering that all samples for individual omics analysis contain a mixture of the same tissue region which allows reliably connecting the layers and information. For future studies and validation, other organs and tissues could be explored and included, especially cancerous tissues which are known to have the most heterogeneity.

The current study illustrates that a tissue pulverization-lyophilization approach is preserving equally reliable genomic, transcriptomic, proteomic, and metabolomic information and could be used as an alternative preanalytical method to commonly employed fresh-frozen tissue division. Furthermore, by employing the PU approach, more homogenous and reliable inter-layer multi-omics information can be obtained because of reduced local tissue heterogeneity otherwise typically coming from adjacent tissue section usage. Additional advantages of the PU approach include easier long-term storage and transportation of the tissue, as once the water is removed via sublimation, this prevents abiotic hydrolysis and ongoing enzymatic reaction. With this, we propose the PU approach as an improved alternative to the currently used fresh-frozen tissue sectioning for bulk multi-omics tissue analysis, especially within cancer research and when the overall tissue amount is limited for multiple fresh-frozen aliquot generation.

## Materials and methods

### Experimental design

Experimental protocols were approved by Tuebingen Regional Council (Regierungspräsidium Tübingen), Germany. All methods were carried out in accordance with relevant guidelines and regulations. All methods are reported in accordance with Animal Research: Reporting of In Vivo Experiments (ARRIVE) guidelines.

Female BALB/c nude 8-week-old mice (CAnN.Cg-Foxn1^nu^/Crl) were purchased from Charles River (Charles River Laboratories, Germany). Mice had ad libitum access to a regular diet (Table S1) and water. After 8 weeks of housing, mice were sacrificed using 2% isoflurane for 5 min and subsequent decapitation. For fast excision and quenching of the target organs, a two-branched workflow was used: brain hemispheres were excised in a cryotome chamber (− 4 °C) while at the same time, first kidney, then liver samples were prepared separately on a sterile, ice-cooled metal plate. The overall time between death and snap-freezing in liquid nitrogen of the last sample ranged between 55 s and 4:20 min with a median of 2:19 min for the brain, 1:58 min for the kidney, and 3:06 min for the liver (Table S2). Animals were randomly selected for FF and PU organ groups as there were no treatment involved in this study. No animals were excluded from the cohort. No blinding was performed.

Fresh-frozen (FF) aliquots of intact liver, brain and kidney were stored in individual cryo-vials and distributed for the different omics techniques. For preparation of the pulverized-lyophilized (PU) tissue powder aliquots, the full liver lobes, left kidneys, and left-brain hemispheres were cryogenically pulverized twice in extra thick, inert polyimide tissue tubes (Covaris tissueTUBE XT, Woburn, MA, USA) using an impact level of 2 for brain, 3 for liver and 4 for kidney (Covaris CP02 cryoPREP, Woburn, USA) and dried in a pre-cooled freeze-dryer overnight (Christ Alpha 2–4, Osterode, Germany). The next day aliquots of approximately 10 mg dry powder were prepared from each sample for subsequent omics analysis. To facilitate the handling of the hygroscopic and easily chargeable dry powder, a deionization vent was used to prevent tissue powder loss (Sartorius Ionizing blower, Goettingen, Germany).

### Genomics and transcriptomics: DNA and RNA isolation for enzymatic methyl-(EM) sequencing and RNA-sequencing

Total RNA and DNA were extracted using the DNA/RNA AllPrep mini kit (Qiagen). Frozen tissue was dissociated using the TissueRuptor (QIAGEN GmbH, Düsseldorf, Germany) for 30 s in 10 volumes of RLT Plus buffer (10 µl of buffer for 1 mg of tissue). Lyophilized tissue powder was centrifuged at 1000 g for 1 min and dissociated using the TissueLyser II for 2 min at 30 Hz in a 2 ml microcentrifuge tube and one Stainless Steel Bead of 5 mm (Qiagen). After dissociation, isolation followed a common pipeline for both materials. Dissociated tissue aliquots of 10 mg were stored at − 80 °C. Prior to isolation, 10 mg of dissociated tissue was diluted in a total volume of 600 µl of RNA lysis buffer RLT Plus at room temperature. Isolation was performed using the manufacturer’s recommendation (1035105 11/2005). Briefly, RLT Plus buffer was not complemented with β-mercaptoethanol, optional DNase digestion was performed using the (Qiagen), optional centrifugation at full speed was performed to limit ethanol carryover and elution was performed using 2 × 30 µl of RNase-free water for RNA and 2 × 100 µl of EB buffer for the DNA.

### Preparation of genomic DNA for EM-sequencing

DNA concentration was determined by Qubit measurement using the DNA 1000 kit (ThermoFisher Scientific, Dreieich, Germany). DNA purity was assessed using the A260/A280 and A260/A230 ratio from the NanoDrop 1000 Spectrophotometer. A260/A280 ratios were in the range of 1.9–2.25 while A260/A230 ratios varied between 0.35–3.91 (see Figure S1).

1000 ng of isolated DNA were sheared to an average length of 450 bp using the Covaris ultrasonicator (Covaris E220 Focused Ultrasonicator—AV, Woburn, MA, USA) and subsequently double size selected with SPRI beads (Beckman Coulter) in a bead: sample ratio of 0.52 for the first size selection and 0.75 for the second size selection. In parallel, 40 ng of unmethylated lambda control DNA (NEB, E7123A) and 2 ng CpG-methylated pUC19 control DNA (NEB, E7122A) were mixed, sheared, and double size selected as described above. Sample concentration and average fragment length were determined using the Qubit HS kit (ThermoFisher Scientific, Dreieich, Germany) and the HS kit on the BioAnalyzer 2100 (Agilent Technologies). For each sample 20 ng of sample and 0.21 ng of combined control DNA (lambda + pUC19) were mixed and used as starting material for Enzymatic Methyl-Seq (EM-seq).

To perform EM-seq on the medium-throughput DNA sequencing platform MGISEQ-2000 instrument (MGI Tech Co., Ltd), we combined the MGIEasy Whole Genome Bisulfite Sequencing (WGBS) Library kit (Cat. No 1000005251, kit version v2) with the NEBNext Enzymatic Methyl-seq Conversion Module (NEB #E7125S, version 3.1_2/20) and performed the library preparation according to the respective protocol except for few adaptations.

In summary, end repair, A-tailing, and adapter ligation (dilution 1:5) as well as cleanup of adapter-ligated DNA were performed according to the MGIEasy WGBS Kit User Manual version B0. After eluting the adapter-ligated DNA in 28ul of NF water, we directly continued with the NEBNext Enzymatic Methyl-seq Conversion Module. Oxidation of 5-methylcytosines and 5-hydroxymethlcytosine catalyzed by the ten-eleven translocation enzyme (TET2), clean-up of TET2 converted DNA, DNA denaturation by sodium hydroxide, deamination of cytosines by apolipoprotein B mRNA editing enzyme, catalytic polypeptide-like (APOBEC), and clean-up of deaminated DNA were performed according to manufacturer’s instructions except for eluting the deaminated DNA in 20ul of NF water instead of NEB elution buffer. Next, deaminated DNA was amplified as described in the MGIEasy WGBS Library Prep Kit User Manual version B0 using 15 cycles of amplification to adjust for the increased insert size of ~ 450 bp. After a bead clean-up of the PCR product, the concentration of the samples was measured using the HS Qubit kit and the fragment length was determined using the HS BioAnalyser kit. For each sample 1 pmol of double-stranded PCR product was digested, then circularized using DNA Rapid Ligase and cleaned by bead clean-up. For each sample, 4 µl of circularized double-stranded DNA were used to make DNA nanoballs according to the protocol. The concentration of the single-stranded nanoballs was measured with the ssDNA Qubit kit (ThermoFisher Scientific, Dreieich, Germany).

### Sequencing and data analysis of EM-seq samples

For each converted EM-seq sample 300 ng of freshly prepared DNA nanoballs were combined with 150 ng of freshly prepared DNA nanoballs from unconverted libraries (non-EM-seq libraries) to circumvent sequencing issues due to skewed base pair distribution. Samples were loaded and sequenced on the MGISEQ-2000 instrument to generate 150 bp paired-end reads. The average genome-wide coverage per sample ranged between ~ ninefold and ~ 15 fold with an average of 10.5-fold across all samples (see Figure S2). Raw read sequences were preprocessed to remove MGI adapter sequences with SeqPurge (ngs-bits version 2020_06) and then with the nf-core methylseq pipeline (version 1.5)^43^ with initial trimming disabled (–skip trimming option) and allowing for more mismatches (–relax mismatches option) due to the long-read length.

### Quality assessment of isolated RNA for transcriptomic analysis

RNA was isolated using the AllPrep DNA/RNA/miRNA Universal Kit from Qiagen according to the manufacturer’s instructions. Details are described above. The RNA concentration was measured using Qubit RNA BR Assay Kit (ThermoFisher Scientific, Dreieich, Germany), and RNA purity was assessed using A_260_/A_280_ and A_260_/A_230_ ratio from the NanoDrop ND-1000 Spectrophotometer (PEQLAB, VWR, Darmstadt, Germany). RNA integrity number (RIN) was estimated using the Fragment Analyzer SS Total RNA kit (Agilent, Waldbronn, Germany) and the Bioanalyzer 2100 (Agilent, Waldbronn, Germany). RIN values > 8.5 were considered to signify an intact RNA, < 6 signified degraded RNA.

### Preparation and sequencing of RNA sequencing libraries

For library preparation, mRNA fraction was enriched using polyA capture from 110 ng of total RNA using the NEBNext Poly(A) mRNA Magnetic Isolation Module (NEB) using the liquid handler Biomek i7 (Beckman). Next, mRNA libraries were prepared using the NEB Next Ultra II Directional RNA Library Prep Kit for Illumina (NEB) according to the manufacturer’s instructions. Library molarity was determined by measuring the library size (approximately 400 bp) using the Bioanalyzer2100 with the High Sensitivity DNA assay and the library concentration (approximately 10 ng/µl) using Qubit Fluorometric Quantitation and dsDNA High sensitivity assay (Thermo Fisher Scientific, Waltham, MA, USA). The libraries were denaturated, diluted to 270 pM, and sequenced as paired-end 100 bp reads on an Illumina NovaSeq6000 (Illumina, San Diego, CA, USA) with a sequencing depth of approximately 25 million clusters per sample.

### Analysis of transcriptomics data

Read quality of RNA-seq data in fastq files was assessed using ngs-bits (v.2019_06), to identify sequencing cycles with low average quality, adaptor contamination, or repetitive sequences from PCR amplification. Reads were aligned using STAR v2.7.3a^44^ to the reference genome GRCm38, and alignment quality was analyzed using ngs-bits (v.2019_06) and visually inspected in the Integrative Genome Viewer (v2.7.2). Raw and normalized read counts for all genes were obtained using Subread (v2.0.0) and edgeR (v3.26.8).

Raw expression values are available for 55′421 genes in 24 samples. Raw gene expression was filtered by demanding a minimum expression value of 1 count per million (cpm) in at least 2 samples. Filtered data contains expression values for 19′842 genes. The distribution of logarithmized cpm-normalized expression values shows similar characteristics over all samples. Based on the filtered data set, samples were investigated with respect to their pairwise similarity. Spearman’s rank correlation coefficient was calculated for each pair of samples. A hierarchical clustering was performed on the resulting similarity values. Differential gene expression analysis was conducted based on the filtered gene expression data set. A statistical model incorporating the group property of samples was tested by fitting a negative binomial distribution using a generalized linear model (GLM) approach. For each gene, gene expression fold changes (log2 fold change) were computed and a statistical test was performed to assess the significance, which is given as raw p-value and adjusted p-value (FDR, obtained by Benjamini–Hochberg procedure).

Genomics and transcriptomics data is made available at the Sequence Read Archive (SRA) with the repository number SUB14264382.

The gene expression deconvolution interactive tool (GEDIT version 2 and 3) was employed for deconvolution of the RNA-seq data^45^. The web-based interface of GEDIT was utilized, for the version 2 using the default parameters and the reference matrix MouseBodyAtlas-Full (PMID: 18,442,421)^46^ (Fig. 3c), and for the version 3 using the parameters set to the minimum entropy, 50 signature genes per cell type, and row scaling adjusted to 0 to optimize the deconvolution process (Supplementary Figure S2l). To construct the reference matrix, the Tabula Muris dataset, a comprehensive resource for single-cell transcriptomics in mouse tissues, was utilized^47^. This dataset was accessed and downloaded using the TabulaMurisSenisData R package. From the downloaded data, both the expression matrix and associated cell metadata were extracted. The cell types were subsequently aggregated to create a reference matrix, retaining the 35 cell types with the highest representation to ensure a robust and representative reference for subsequent analyses.

Transcriptomics dataset pathway analysis was performed using an enrichment analysis of Gene Ontology (GO) biological processes utilizing ShinyGO v0.81 (https://bioinformatics.sdstate.edu/go/). For the enrichment analysis, no background gene set was specified, and an FDR < 0.01 and |logFC|> 1. Enrichment analysis was conducted separately for each organ under investigation, focusing on the GO Biological Process category.

### Proteomics

For proteomics, a comparison between direct tissue homogenization in the Precellys 24 (Bertin Instruments, Montigny-le-Bretonneux, France) system and homogenization with prior pulverization in the Covaris (Covaris CP02 cryoPREP, Woburn, USA) system was performed for four biological replicates and three mouse tissue types (brain, kidney, and liver).

Per mg fresh tissue biopsy 6 µL of 85% methanol (chilled) and for the hygroscopic powdered tissue 100–200 µL (depending on the sample amount) was added to Precellys vials (Soft tissue CK14 0.5 mL, Bertin Instruments, Montigny-le-Bretonneux, France) and homogenized 3 times for 20 s at 6500 rpm. In between the three rounds, samples were chilled for 30 s on ice. Afterwards samples were centrifuged (10 min, 10,000 g, 4 °C) and supernatants were removed. Pellet and ceramic beads were mixed with 100 µL lysis buffer containing 6 M urea and 0.1 M ammonium bicarbonate and homogenized for 20 s at 6500 rpm. After centrifugation (10 min, 10,000 g, 4 °C) protein content of the supernatant was determined by using the Bradford assay (Bio-Rad Protein Assay Dye Reagent Concentrate, Bio-Rad, Hercules, California, USA) according to the manufacturer’s protocol for microtiter plates.

For the in-solution-digest, a volume of the supernatant corresponding to 10 µg of total protein for each sample was used and proteins were digested according to a previously published protocol^48^ with minor modifications (no heat was applied after the addition of 0.1 M DTT, digestion time 2 h, stop of the reaction with 1.9 µL 100% TFA, adapted incubation times). In the last step, the lower phase was recovered and purified using StageTips (Thermo Fisher Scientific) according to the manufacturer’s protocol. The eluate was concentrated to a final volume of 5 µL and stored at − 20 °C prior to analysis.

For injection, samples were diluted to a final concentration of 10 µL in 0.5% TFA. Global bottom-up proteomics was performed on an Ultimate 3000 RSLC nano-LC coupled to Orbitrap Fusion Tribrid mass spectrometer (Thermo Fischer Scientific) as described before^49^. Data were analysed using Maxquant data analysis algorithm (Version1.6.17.0)^50^. Spectra were matched against Uniprot entries for *Mus musculus* (05.2023 version, 17,807 entries). Fragment ion mass tolerance was set to 0.5 Da and parent ion tolerance to 20 ppm. Carboxymethylation of cysteine was set as fixed modification and methionine oxidation, asparagine and glutamine deamidation as variable modifications. Only proteins identified with a minimum of 1 unique peptide, and an FDR < 0.01 on proteins and peptides levels, and in at least 70% of biological replicates per group were retained for statistical analysis.

Proteins that contained similar peptides and could not be differentiated based on MS/MS analysis alone were grouped to satisfy the principles of parsimony. Proteomics data is made available on the MassIVE database under the reference code MSV000094288 (https://massive.ucsd.edu/ProteoSAFe/dataset.jsp?task=fc4ef0e54fac4117ae63944375e60ed6), and referenced in the ProteomeXchange data repository by the dataset identifier PXD050521 (https://proteomecentral.proteomexchange.org/cgi/GetDataset?ID=PXD050521).

### Metabolomics: NMR spectroscopy-based metabolomics sample preparation, measurements, and data processing

Metabolites were extracted from the pulverized-lyophilized (PU) dry powder and fresh-frozen (FF) tissue using a 2-phase extraction protocol with a mixture of methanol, methyl *tert*-butyl ether (MTBE), and ultrapure water with the focused ultrasonication technology (Covaris E220 evolution Focused Ultrasonicator, Woburn, MA, USA). After two-phase separation, the aqueous layer was evaporated to dryness.

For NMR spectroscopy-based metabolomics analysis, dried metabolite pellets were resuspended in 60 µL of deuterated phosphate buffer (200 mM K_2_HPO_4_, 200 µM NaN_3_, pH 7.4) containing 1 mM of the internal standard 3-(trimethylsilyl) propionic-2,2,3,3-d_4_ acid sodium salt (TSP). To obtain a maximum dissolution, the plastic tubes were thoroughly vortexed and then centrifuged for 5 min at 14,000 × g. 45 µL of the clear supernatant were transferred into 1.7 mm NMR tubes and a 96-tube rack was placed into the cooled (6 °C) NMR autosampler (Bruker BioSpin, Ettlingen, Germany).

Spectra were recorded on a 600 MHz ultra-shielded NMR spectrometer (Avance III HD, Bruker BioSpin, Ettlingen, Germany) equipped with an ultra-sensitive 1.7 mm triple resonance (^1^H, ^13^C, ^31^P) room temperature probe operating at 298 K. A Carr-Purcell-Meiboom-Gill (CPMG) 58 min 48 s experiment was performed including the suppression of water and residual macromolecule background signals (time domain—64 k points, sweep width—20 ppm, 512 scans).

The recorded free induction decay (FID) was Fourier-transformed and spectra properly phase- and baseline-corrected (Bruker Topspin 3.6.1, Ettlingen, Germany). Metabolite annotation and quantification were performed with ChenomX NMR Suite 8.5 by creating an individual library for brain, kidney, and liver metabolites, considering ChenomX software commercial metabolite annotation library, human metabolome database (HMDB), and available reference literature. Preliminary statistical analysis was performed using MetaboAnalyst 5.0 and 6.0 platforms (https://www.metaboanalyst.ca/)^51^.

Metabolomics data have been deposited to the EMBL-EBI MetaboLights^52^ data repository with the identifier MTBLS12137.

### Metabolomics: LC–MS-based metabolomics and the quenching efficiency

Extraction of the FF tissue samples was performed similar to a published method^53^. Frozen tissue samples were homogenized in a cold (− 20 °C) mixture of methanol and water (3.2:1, v/v) in a FastPrep-24™ instrument equipped with a CoolPrepTM adapter (MP Biomedicals, Heidelberg, Germany). Homogenization was achieved in Lysing matrix Z tubes at a final ratio of 15 µl solvent/mg wet tissue.

The PU dry powder tissue was extracted similarly in a Bioruptor® sonication system (Diagenode, Liège, Belgium) using a cold (− 20 °C) mixture of methanol and water (3:1, v/v) at a final ratio of 50 µl solvent/mg dry tissue. Homogenates were centrifuged and aliquots of the supernatants used for metabolite analysis with LC–MS–MS. Quantification of adenosine phosphates AMP, ADP and ATP was performed as described previously^54^. Intermediates of glycolysis and pentose phosphate pathway (hexose-6-phosphate, phosphoenolpyruvate, 2-/3-phosphoglycerate, fructose-1,6-bisphosphate, 6-phosphogluconate, sedoheptulose-7-phosphate, ribose-5-phosphate, and ribulose-5-phosphate/xylulose-5-phosphate) were determined similar to a described method^55^ using (A) water:acetonitrile 87.5:12.5 (v/v), and (B) water:acetonitrile 50:50 (v/v), each with 750 mg/l octylammonium acetate, as mobile phases.

### Statistical analysis

Principal component analysis (PCA) displays the first principal component on X axis, and second on the Y axis, with 95% confidence interval cloud colored around individual sample points. Pearson r distance measure was used for correlation heatmaps with auto scaled color distribution. For hierarchical clustering dendrogram, distance was calculated by the Euclidean distance measure using Ward clustering algorithm. No statistical significance was observed for any one-way ANOVA analysis between the individual tissue preparation method comparisons. Error bars represent the mean and standard error of the mean (SEM).

## Electronic supplementary material

Below is the link to the electronic supplementary material.

Supplementary Material 1

Supplementary Material 2

## Data Availability

Genomics and transcriptomics data are submitted to the Sequence Read Archive (SRA) with the repository number SUB14264382. Proteomics data is made available on the MassIVE database under the reference code MSV000094288 https://massive.ucsd.edu/ProteoSAFe/dataset.jsp?task=fc4ef0e54fac4117ae63944375e60ed6, and referenced in the ProteomeXchange data repository dataset identifier PXD050521 https://proteomecentral.proteomexchange.org/cgi/GetDataset?ID=PXD050521. NMR spectroscopy-based metabolomics data have been deposited to the EMBL-EBI MetaboLights data repository with the identifier MTBLS12137, https://www.ebi.ac.uk/metabolights/ MTBLS12137. Targeted LC–MS dataset is available as a Supporting Dataset S1.
