# Supplementary material for: Cryogenic mouse tissue homogenization as an alternative to fresh-frozen biopsy use for genomics, transcriptomics, proteomics and metabolomics
Source: Sci Rep. 2025 Jun 23;15:20254. doi: 10.1038/s41598-025-06438-3 (PMC12185734; doi:10.1038/s41598-025-06438-3)
Supplement: Supplementary file 1 — Supplementary Material 1 [file 41598_2025_6438_MOESM1_ESM.docx]

Supplementary information

Cryogenic mouse tissue homogenization as an alternative to fresh-frozen biopsy use for genomics, transcriptomics, proteomics and metabolomics

Laimdota Zizmare^1,2^, Ute Hofmann^3^, Mohamed Ali Jarboui^4^, Franziska Klose^4^, Sabine Fraschka^5,6^, Jakob Matthes^5,6^, Marcel Krüger^1^, Elke Schaeffeler^2,3^, Matthias Schwab^2,3,7^, Marius Ueffing^4^, Bernd J. Pichler^1,2^, Karsten Boldt^4^, Nicolas Casadei^5,6^, Christoph Trautwein^1,2,8,*^

^1^ Werner Siemens Imaging Center, Department of Preclinical Imaging and Radiopharmacy, University of Tübingen, 72076 Tübingen, Germany.

^2^ Cluster of Excellence iFIT (EXC2180) "Image-Guided and Functionally Instructed Tumor Therapies", University of Tübingen, 72076 Tübingen, Germany.

^3^ Dr. Margarete Fischer-Bosch Institute of Clinical Pharmacology and University of Tübingen, 70376 Stuttgart, Germany.

^4^ Core Facility for Medical Proteomics, Institute for Ophthalmic Research, University of Tübingen, 72076 Tübingen, Germany.

^5^ NGS Competence Center Tübingen (NCCT), Institute of Medical Genetics and Applied Genomics, University of Tübingen, 72076 Tübingen, Germany.

^6^ Institute of Medical Genetics and Applied Genomics, University of Tübingen, 72076 Tübingen, Germany.

^7^ Department of Clinical Pharmacology, and Department of Biochemistry and Pharmacy, University of Tübingen, 72076 Tübingen, Germany.

^8^ Core Facility Metabolomics, Faculty of Medicine, University of Tübingen, 72076 Tübingen, Germany.

*Corresponding author, email: Christoph.Trautwein@med.uni-tuebingen.de

**This PDF file includes:**

Figures S1 to S4

Tables S1 to S5

**Other supporting materials for this manuscript include the following:**

Dataset S1


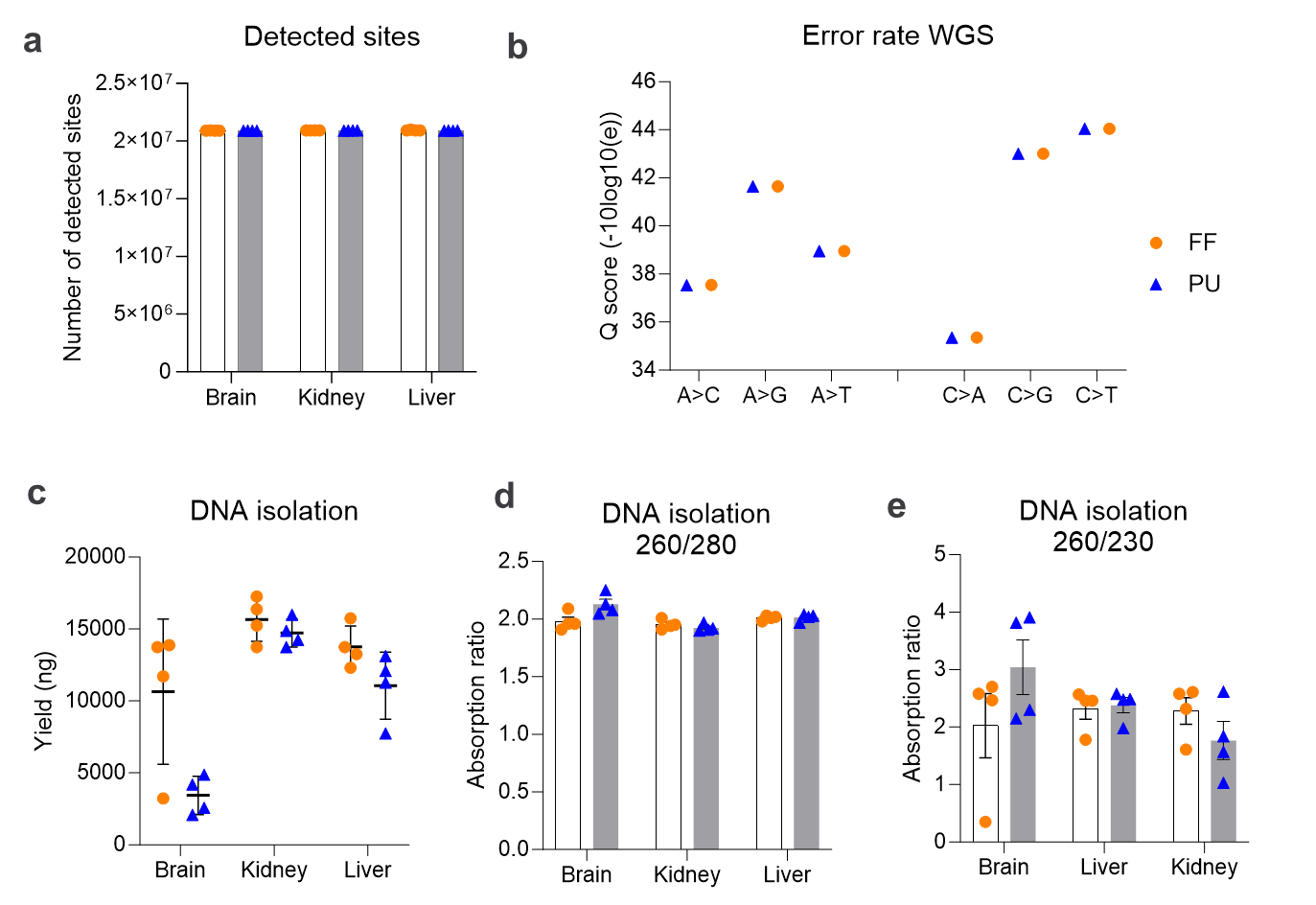


Fig. S1. Whole-genome sequencing and enzymatic methyl-sequencing (EM-seq) illustrates reliably-preserved genome coverage. One sample from FF and PU was subjected to whole-genome sequencing (WGS) for quality control, and example comparable (a) number of detected sited and (b) error rates Q score. Further, DNA isolation was verified by (c) DNA isolation yield, (d) DNA isolation based on 260/280 and (e) 260/230, showing comparable sample quality for both FF and PU tissue processing. FF- fresh-frozen, orange dot; PU – pulverized-lyophilized, blue triangle. Error bars represent the mean and standard error of the mean (SEM).


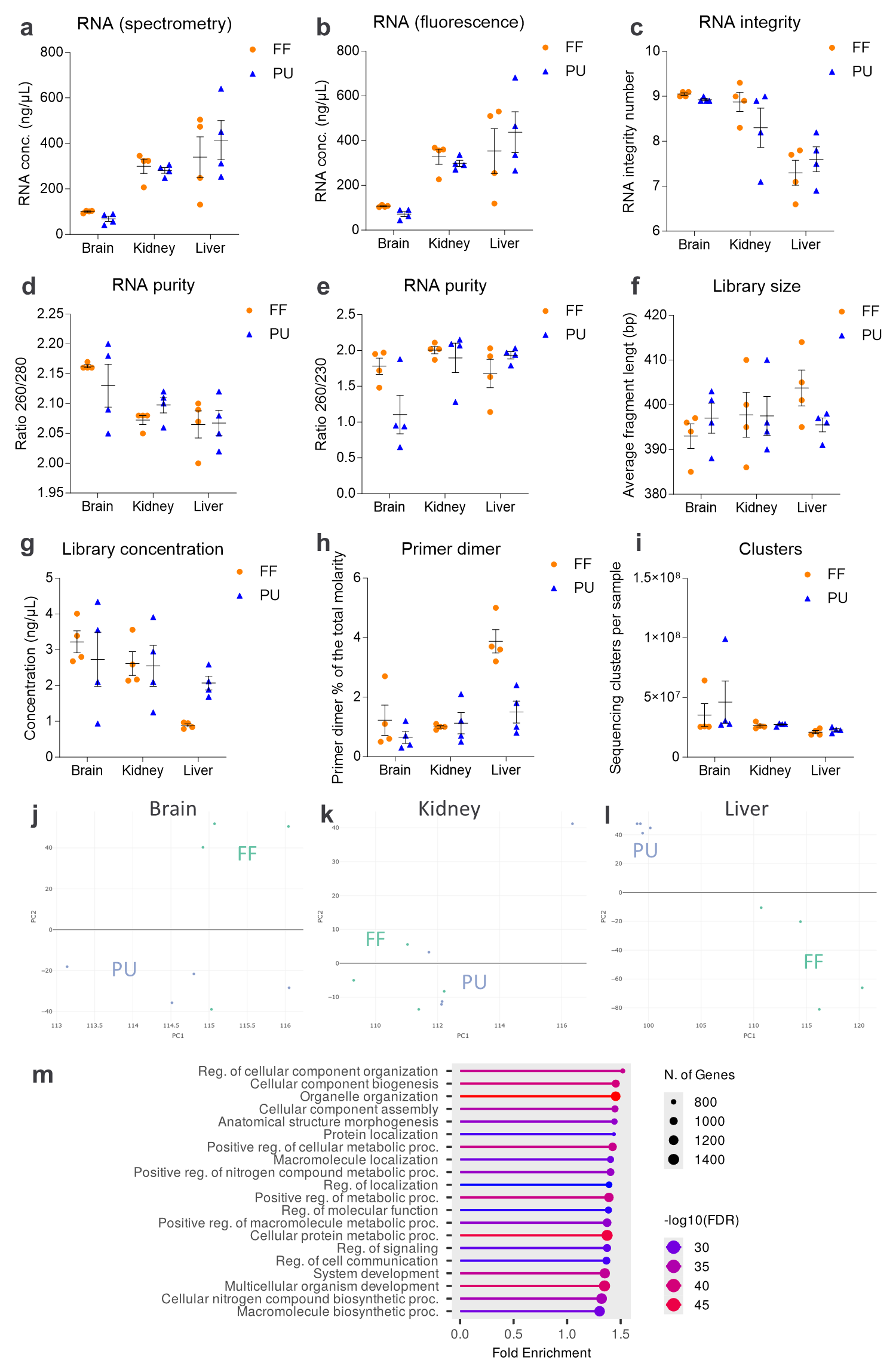


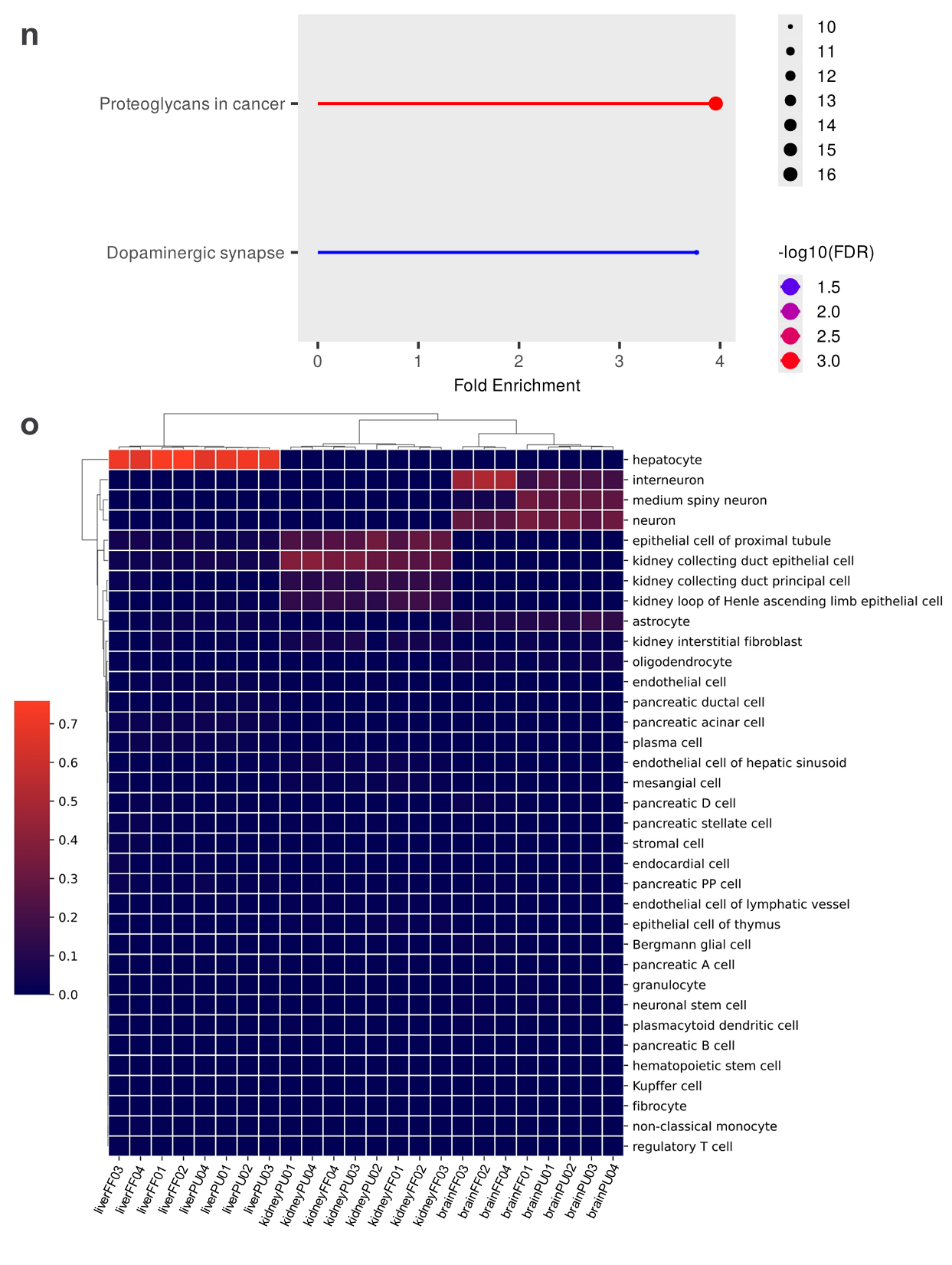


Fig. S2. RNA sequencing quality control and the impact of tissue preparation method on the performance of library preparation. (a) Spectrometry (NanoDrop) and (b) fluorescence (Qubit) analysis validate obtained RNA concentrations as comparable between the fresh-frozen (FF) and pulverization (PU) tissue processing methods, and tissue-type dependent. (c) Obtained RNA integrity number (RIN) illustrates similar RNA integrity by FF and PU tissue preparation protocols. RNA purity at (d) 260/280 and (e) 260/230 ratios are comparable between the FF and PU methods. Both fresh frozen (FF) and pulverized (PU) tissue preparation methods yielded similar (f) library size, (g) library concentrations, (h) percentage of primer dimers in the samples (in exception for the liver tissue, where the FF method yielded increased primer dimer %); and (i) sequencing clusters per sample. Individual principal component analysis for (j) brain, (k) kidney, and (l) liver. Pathway enrichment analysis of gene ontology biological processes for (m) liver and (n) brain tissue associated with PU vs FF altered transcriptome changes. (o) The gene expression deconvolution with comprehensive resource for single-cell transcriptomics in mouse tissues for liver, kidney and brain cell subtype alterations by FF and PU.


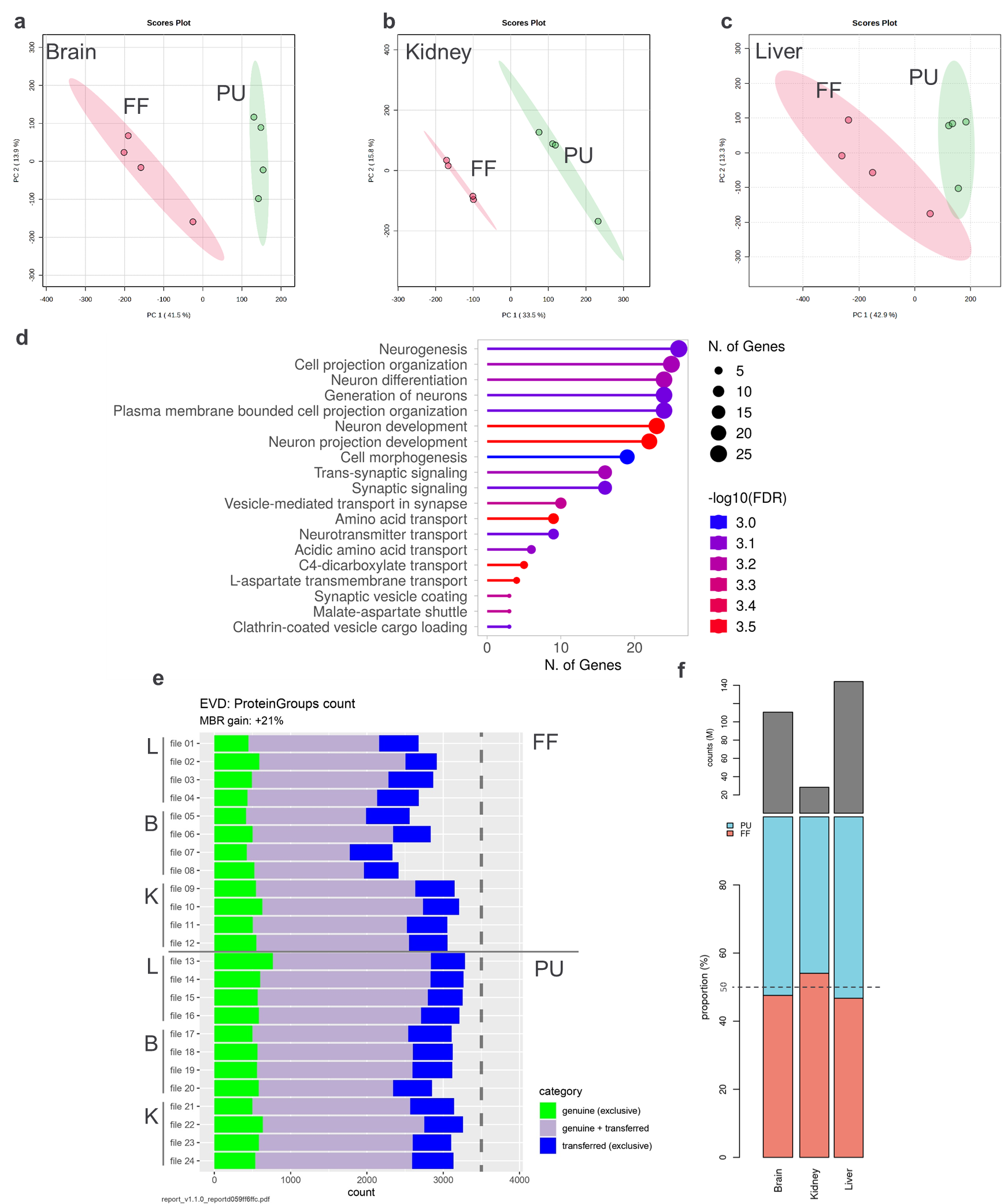


Fig. S3. Proteomics analysis. Principal component analysis of all three tissues illustrating tissue-specific clusters for individual (a) brain, (b) kidney, and (c) liver tissue proteome reveal reduced protein variability in pulverized-lyophilized (PU) tissue compared to fresh-frozen sliced (FF) brain and liver tissue. (d) The elucidation of all tissue type combined analysis of FF method impact reveals mainly affected neuronal function-related pathways based on GO biological process pathway database. (e) Protein group count represented for individual samples, and (f) comparing protein proportion origin from PU and FF approaches in each tissue type.


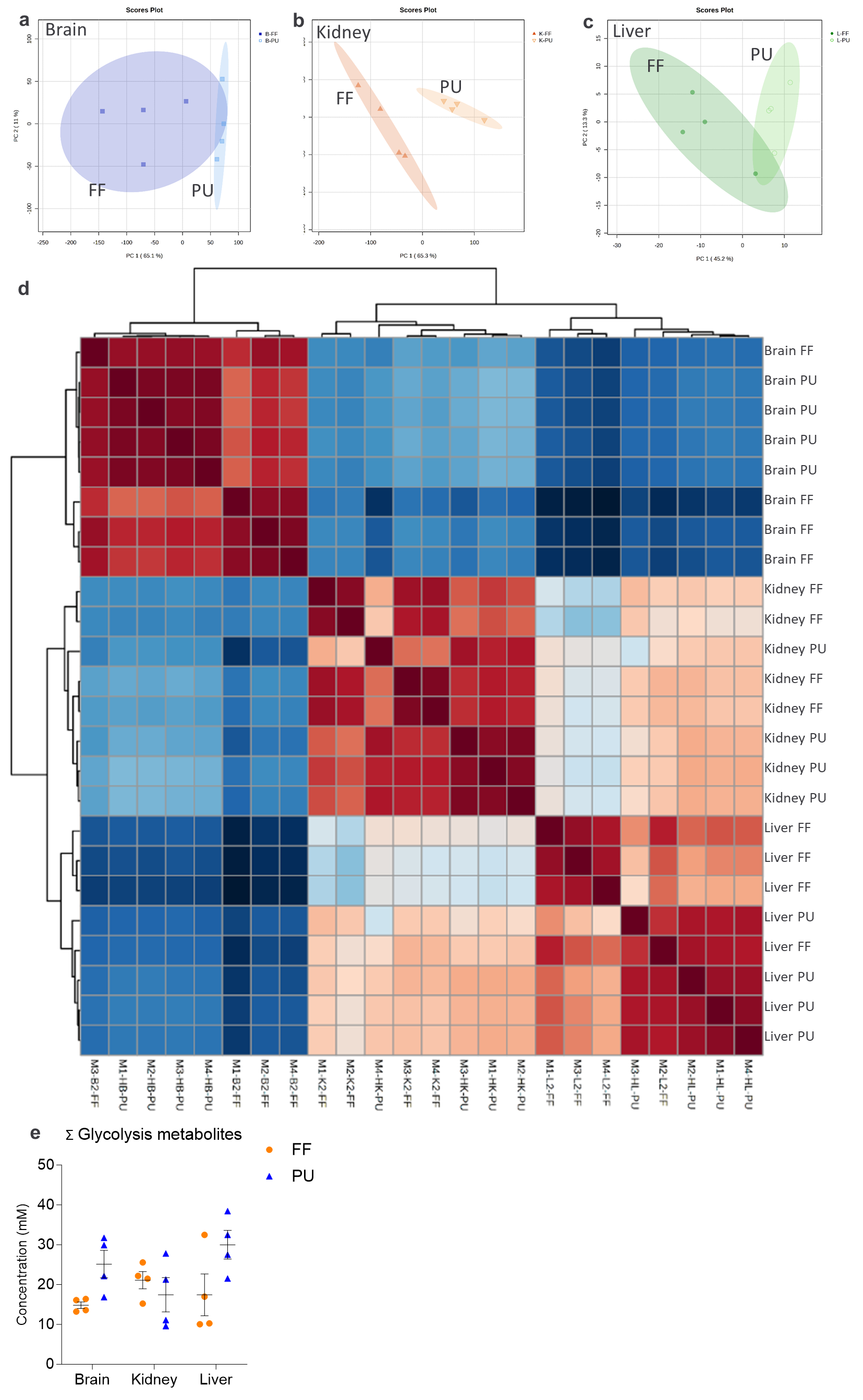


Fig. S4. Metabolomics analysis. Principal component analysis of NMR spectroscopy-based metabolite quantification in the (a) brain, (b) kidney, and (c) liver tissue metabolome reveal reduced protein variability in pulverized-lyophilized (PU) tissue compared to fresh-frozen sliced (FF) tissue. (d) NMR spectroscopy-based metabolomics sample correlation analysis indicates tissue type correlation and further PU tissue similarity. (e) LC-MS-based metabolite identification revealed relatively higher preservation of glycolysis metabolites in brain and liver PU compared to FF tissue, while the kidney shows a comparable sum of glycolysis metabolites with both tissue preparation setups.

Table S1. Mouse diet. V1534-703 10 mm, sterilized (γ-irradiated with 25 kGy), low energy content, low in nitrosamines, well suited for long-term studies (Herstellung und Vertrieb ssniff Spezialdiäten GmbH, Germany).

| Gross energy (GE) | 16.2 MJ/kg |
| --- | --- |
| Convertible energy (ME) 1) | 13.5 MJ/kg |
|  | |
| **Nutrients** | **[%]** |
| Protein (N x 6.25) | 19.0 |
| Fat | 3.3 |
| Fiber  NDF  ADF | 4.9  17.2  7.1 |
| Ash | 6.4 |
| Starch | 35.2 |
| Sugar | 5.3 |
| N free extractives | 54.2 |

Table S2. Multi-omics tissue collection work plan: timing and tissue weights. Four mice (M) were weighted, sacrificed, the collected organs were weighted and quenching was timed.

|  | Full body weight [g] | Time until quenching | Wet weight [mg] | Time until quenching | Wet weight [mg] | Time until quenching | Wet weight [mg] | Time until quenching | Wet weight [mg] |
| --- | --- | --- | --- | --- | --- | --- | --- | --- | --- |
|  | Weight | Liver big lobe |  | Liver lobe 1 |  | Liver lobe 2 |  | Liver lobe 3 |  |
| M1 | 25.2 | 02:55 | 337 | 03:30 | 171 | 03:55 | 105 | 04:20 | 99 |
| M2 | 24.8 | 02:46 | 321 | 03:08 | 101 | 03:25 | 178 | 03:30 | 98 |
| M3 | 26.1 | 02:53 | 355 | 03:05 | 209 | 03:12 | 118 | 03:19 | 122 |
| M4 | 26.1 | 02:40 | 357 | 02:50 | 237 | 02:55 | 121 | 03:00 | 143 |
|  | | | | | | | | | |
|  | Weight | Left Kidney |  | Right Kidney 1 |  | Right Kidney 2 |  | Right Kidney 3 |  |
| M1 | 25.2 | 01:57 | 177 | 02:45 | 51 | 03:01 | 66 | 03:17 | 80 |
| M2 | 24.8 | 01:13 | 194 | 01:55 | 61 | 02:01 | 59 | 02:04 | 77 |
| M3 | 26.1 | 00:55 | 188 | 01:43 | 78 | 02:07 | 85 | 02:13 | 55 |
| M4 | 26.1 | 01:08 | 211 | 01:45 | 80 | 01:52 | 47 | 01:59 | 98 |
|  | | | | | | | | | |
|  | Weight | Left Brain |  | Right Brain 1 |  | Right Brain 2 |  | Right Brain 3 |  |
| M1 | 25.2 | 02:08 | 213 | 02:26 | 44 | 02:49 | 54 | 03:00 | 49 |
| M2 | 24.8 | 02:01 | 159 | 02:18 | 61 | 02:29 | 72 | 02:40 | 94 |
| M3 | 26.1 | 01:45 | 130 | 02:01 | 79 | 02:20 | 56 | 02:28 | 66 |
| M4 | 26.1 | 01:38 | 169 | 01:52 | 62 | 02:06 | 85 | 02:20 | 85 |

Table S3. Transcriptomics pathway analysis of the PU versus FF tissue at |FC|>1 and FDR<0.05. Brain and liver transcripts led to differently enriched 178 pathways. Highlighted in box similarly enriched pathways between the brain and the liver.

Table S4. Transcriptomics pathway analysis for the |FC| > 1 and p < 0.01 threshold. Only 7 pathways were enriched by all tissue type transcripts. The most prominent enrichment was for the CREB signaling in neurons. This pathway was however positively enriched in brain and the liver, while negatively enriched in the kidney. Overall transcriptomics analysis and pathway enrichment indicated no specific trend for fresh-frozen versus pulverized-lyophilized tissue processing method that would lead to superior pathway presentation or enrichment.

Table S5. Proteomics pathway analysis of the FFvsPU tissue at FDR <= 0.05 and a FC>1. Overview representing Venn diagram, and full table showing brain, kidney, and liver proteome 33 altered pathways.


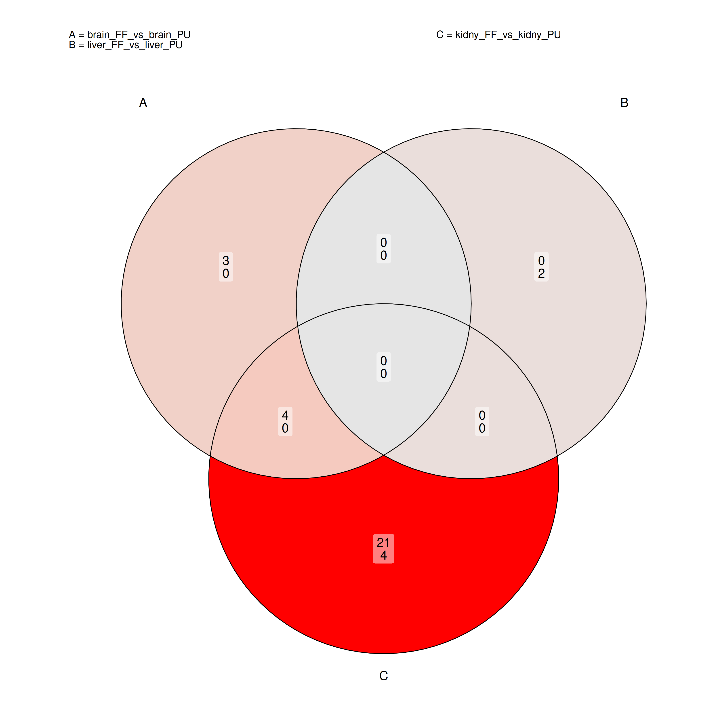

Dataset S1 (separate file). Targeted LC-MS dataset.
